# Statistics of protein-DNA binding and the total number of binding sites for a transcription factor in the mammalian genome

**DOI:** 10.1186/1471-2164-11-S1-S12

**Published:** 2010-02-10

**Authors:** Vladimir A Kuznetsov, Onkar Singh, Piroon Jenjaroenpun

**Affiliations:** 1 Department of Genome and Gene Expression Data Analysis, Bioinformatics Institute, 30 Biopolis str #07-01, Singapore, 138671; 2 Laboratory of Clinical Pharmacology, Division of Medical Sciences, National Cancer Centre, 11 Hospital Drive, Singapore 169610

## Abstract

**Background:**

Transcription factor (TF)-DNA binding loci are explored by analyzing massive datasets generated with application of Chromatin Immuno-Precipitation (ChIP)-based high-throughput sequencing technologies. These datasets suffer from a bias in the information about binding loci availability, sample incompleteness and diverse sources of technical and biological noises. Therefore adequate mathematical models of ChIP-based high-throughput assay(s) and statistical tools are required for a robust identification of specific and reliable TF binding sites (TFBS), a precise characterization of TFBS avidity distribution and a plausible estimation the total number of specific TFBS for a given TF in the genome for a given cell type.

**Results:**

We developed an exploratory mixture probabilistic model for a specific and non-specific transcription factor-DNA (TF-DNA) binding. Within ChiP-seq data sets, the statistics of specific and non-specific DNA-protein binding is defined by a mixture of sample size-dependent skewed functions described by Kolmogorov-Waring (K-W) function (Kuznetsov, 2003) and exponential function, respectively. Using available Chip-seq data for eleven TFs, essential for self-maintenance and differentiation of mouse embryonic stem cells (SC) (Nanog, Oct4, sox2, KLf4, STAT3, E2F1, Tcfcp211, ZFX, n-Myc, c-Myc and Essrb) reported in Chen et al (2008), we estimated (i) the specificity and the sensitivity of the ChiP-seq binding assays and (ii) the number of specific but not identified in the current experiments binding sites (BSs) in the genome of mouse embryonic stem cells. Motif finding analysis applied to the identified c-Myc TFBSs supports our results and allowed us to predict many novel c-Myc target genes.

**Conclusion:**

We provide a novel methodology of estimating the specificity and the sensitivity of TF-DNA binding in massively paralleled ChIP sequencing (ChIP-seq) binding assay. Goodness-of fit analysis of K-W functions suggests that a large fraction of low- and moderate- avidity TFBSs cannot be identified by the ChIP-based methods. Thus the task to identify the binding sensitivity of a TF cannot be technically resolved yet by current ChIP-seq, compared to former experimental techniques. Considering our improvement in measuring the sensitivity and the specificity of the TFs obtained from the ChIP-seq data, the models of transcriptional regulatory networks in embryonic cells and other cell types derived from the given ChIp-seq data should be carefully revised.

## Background

Identification of transcription regulatory elements in the genome is an important problem of molecular systems biology and statistical genomic studies. Among those elements, transcription factor binding sites (TFBSs), short and specific DNA loci targeted by transcription factors (TFs), are considered as basic regulatory elements of gene functional activity and reflect the corresponding protein-DNA interactions in a cell. TFs are the largest set of regulatory proteins in mammalian cells. According to NCBI RefSeq database, about 10% of all known proteins of mammals, including humans, are TFs.

A TFBS serves as a target for a transcription factor which binds to this specific binding site (BS) directly or via other proteins and regulates gene transcription. In a mammalian genome the number of direct and indirect BSs for a given TF could be ranged from several hundreds to hundred thousand [1-8]. However, these values have not been directly measured and the theoretical estimates provide lowly confident values.

The interactions between the molecules of a given TF and corresponding TFBSs in the genome could be considered in the terms of TF-DNA binding events (BEs) which reflect the events of binding in the assay. Any of such events might be specific and non-specific in the context of a direct physical binding of the TF to a TFBS. The intensity (and the corresponding probability) of an occurrence of a given BE in a given genome locus can be characterized by the level of relative avidity (RA) of the TF-DNA binding- an integrative quantitative characteristic of availability of a DNA locus (e.g. TFBS and its flanking region) for a given protein (e.g. TF) binding [9].

The population distribution function of RA (i.e. the distribution function of RA for a given set (population) of BE) for a given TF can reveal functional attributes of the TFBSs and the mechanisms of the TF-DNA interaction on the genomic scale. However, at the level of single cells or cell samples the distribution function of RA for any TF is unknown, since many technical problems of direct counting of specific protein molecules bound DNA have not been solved yet. Instead, the relative avidity of TF-DNA binding in an average genome within a given cell sample can be quantified by an estimate of the number of TF molecules bound to a given locus averaging across the given cell sample. However, quantitative detection of TFs bound to specific loci is a great challenge. A simpler task is a pull-down of short DNA fragments directly or indirectly bound with the molecules of a given TF. Such TF-DNA complexes can be detected in Chromatin Immuno-Precipitation (ChIP) ChIP-based genome-wide sequencing experiments [3,4,6-8].

In a typical genome-scale ChIP experiment ~10^8 ^cells are used and the transcription factors are cross-linked to their DNA using chemical cross linkers. After the genomic DNA is isolated and fragmented by ultrasound sonication, an antibody specific to the TF of interest is used to isolate each TF molecule and the DNA fragment which it is bound to. Cloning the whole pool of such fragments and sequencing them (for example, in a serial analysis of chromatin occupancy (SACO) [10], ChIP-paired end tag (ChIP-PET) [7], sequence tag analysis of genome enrichment (STAGE) [1]) provides the data for a large-scale identification of TFBSs.

However, the experimental information, obtained from SACO, ChIP-PET and STAGE experiments, about statistical properties of TF binding at specific physical BSs (and moreover biological functions of the BSs) in a given cell type, at a given environment is highly noisy and essentially incomplete. Only pulled-down TF-DNA complexes containing DNA fragments with maximal relative binding avidity could be reliably detected.

**Figure 1:**
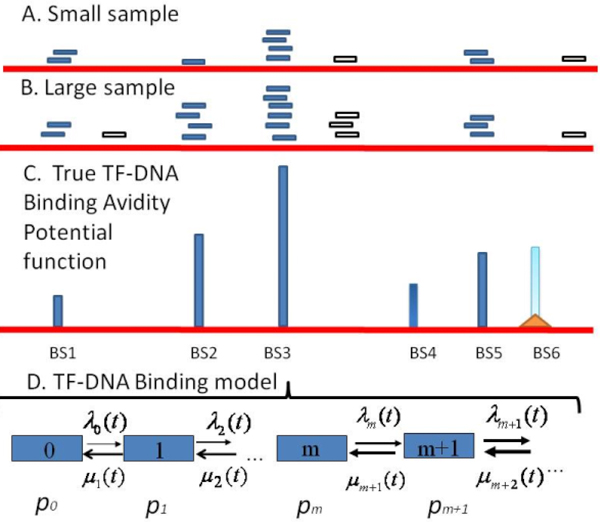
**A random sampling model of determination of TF binding avidity potential on the genome defined in a ChIP-seq experiment**. Sequencing TFBS-enriched DNA fragments can be assayed to determine the specific clusters of DNA sequences bound by TF protein. Results are strongly depended from the number of read (sample size). A: Small sample size. B: Large sample size. Blue horizontal stake: specific DNA fragment; white horizontal stake: non-specific DNA fragment forming non-specific (false-positive) clusters. C: BS1-BS6 are binding loci presented in the given cells: blue vertical stakes are relative binding avidity in the loci; BS6 might be modified (epigenetically) and suppressed BS (a stake with triangle basis) and therefore it might be not detected in ChIp-seq assay. BS1 and BS4 might be not detected in the assay due to sample size limit. D: A scheme of the random Markov process of binding-dissociation of TF-DNA realized on the genome scale. The graph illustrates concept of birth-death random process model utilized in this work (see Methods).

Recently, a new generation of sequencing technology, massively parallel sequencing (MPS), has been established [2,5] MPS can sequence many millions of fragments in a single experiment. Combining ChIP and MPS (ChIP-seq) provides a genome wide view on TFBSs. In particular, ChIP-seq method can accurately detect moderate/high avidity TFBSs at a resolution (up to several base pairs) higher than for any previous ChIP method [2,5]. In detail, during a ChIP-seq experiment, TF-Immuno-precipitated DNA fragments (sequence reads) are directly sequenced in series of 25-36 bp reads, and dozen millions of such short reads are then mapped to the reference genome. After mapping of unique DNA fragments (enriched by DNA bound by TF molecules) onto the genome, the DNA fragment sequences, extended further up to ~200 bp, are clustered by their overlapping sub-sequences and mapped onto the genome. The number of DNA fragment sequence overlaps is further represented in the analysis as overlap signal with peaks. Each DNA fragment cluster is usually quantified by its highest overlap peak, i.e. by the cluster's internal region with the largest number of overlapping extended ChIP-seq DNA fragment sequences. (Figure 1) The number of overlapping DNA fragments in a given cluster can be observed as the RA value of the given TF-DNA BS. Technically, TF binding avidity in specific DNA loci could be measured on the genome scale using peak search procedure on the mapped overlap signal. A number of software tools have been proposed to perform this search in the genome, and thus to find genomic regions containing the TF-abundant DNA loci and to identify in each locus specific TF-bound DNA sequences.

One of the crucial problems with ChIP-based genome-wide assays, including ChIP-seq, is a statistically reliable identification of biologically meaningful phenomena (e.g. all specific and functionally important binding loci) from the large amounts of generated experimental data. In this context reliable, specific and sensitive mapping of protein-DNA interactions is still essentially dependent on subjective rules of pre-processing and filtration of the DNA fragment sequences, the statistical criteria used to identify specific binding loci and the real TFBSs. As a result, some  basic definitions, datasets, statistical models  and estimates have been revised after  original publications [2,5,9,11-13].

A large unexplained "technical" noise component in the experimental measurements and its non-uniform location in the genome are serious limitations for the specificity of the current ChIP-based methods [7,9,11-13]. Comparison of ChIP-seq data sets analysing the same TF, under similar conditions and the same cell type but using different experimental platforms (ChIP-chip, ChIP-PET and ChIP-seq) revealed a reasonable consistency between the mapped datasets only for high and moderate avidity ChIP-defined binding loci. These loci, however, represent only a small fraction of all binding loci observed in the course of the entire ChIP-seq experiment. Therefore genomic localization and biological roles of other reliable but less reactive TF-binding loci (low avidity BSs) cannot be identified from the assays [9,11,12,14]. To analyse statistical properties of the low-, moderate- and high-avidity binding loci for a given ChIP-seq data set we need to develop a probabilistic model which would allow us to answer three important open questions.

How many 'hidden' TF-specific BSs with moderate and low avidity are present in the noise-rich subset of ChIP-seq data?

How many "true negative" specific BSs which should be included in consideration are not present in the given ChIP-seq dataset?

How many specific BSs of a given TF exist in the genome?

To answer these questions an analytical model(s) of ChIP-seq TF-DNA binding experiments should be focused on analysis of *specificity *and *sensitivity *of protein-DNA binding.

To do this a simple exploratory probabilistic model of TF-DNA binding events was developed and implemented. It allowed us to facilitate noise filtering of ChIP-seq datasets and predicting, for a given TF, the number of specific TF-DNA binding loci in the entire genome. We applied the proposed analytical approach to the distributions of binding avidity of the TFBS of eleven TFs which are essential for maintaining and differentiation mouse embryonic stem cells.

## Results

### Natural variation of binding availability of the TFBS for TFs and critical threshold of TF binding specificity

TFs bind to short high affinity DNA response elements (motifs) mostly in putative promoter region of a gene and modify gene activity. However this model of TF-DNA binding is too simple and the phenomenon remains poorly understood, although many models of TF-DNA binding have been reported [9,13,15-17]. For example, well-known TF c-Myc exhibits very diverse and complex patterns of DNA binding activity [12]. C-Myc binding region in gene promoter region could often contain multiple copies of a few c-Myc E-box sequences. Such TFBS clusters could be found in low-, moderate- and high- avidity ChIP-seq binding loci of the mouse embryonic stem cell (EC) -related genes (see below).

The E-box is a high affinity response/binding element of DNA which able to discriminate c-Myc among other different TFs. c-Myc forming heterodimers with its binding partners (MAX, MIZ1) and other DNA-specific proteins (e.g. MIZ1, Sp1, NEY) using "canonical" E box 5'-CACGTG-3', which represents the consensus or other at least five "non-canonical" E-boxes (CATGTG, CATGCG, CACGCG, CACGAG, CAACGTG), and additional one (CGCGAG) which we report in this work (see below). The E-box motifs are present in putative promoter regions of at least 4000 genes [12]; however, how a given response element can discriminate among all these different transcription factors is currently unclear. Moreover, several types of non-canonical E-boxes could bind c-Myc-MAX complex strongly than canonical [18]. In this respect, several mechanisms of variability of c-Myc-Max-DNA binding avidity have been proposed (and, in some instances, experimentally substantiated) [12,17,18]. For instance, flanking sequences, chromatin structure, methylation status, and relative position within the promoter or interaction with adjacent regulatory elements might contribute to selection of a particular complex [17].

We assume here that transcription factor (TF)-DNA binding avidity in a given TFBS defined in ChIP-seq experiment could be considered as useful integrative characteristic of availability of the TFBS for a given TF. Naturally, TF-DNA binding avidity could be quantified by the number of TF molecules binding a given specific locus including specific binding element(s) and its flanking regions. However, the number of TF molecules bound DNA cannot be directly inferred from ChIP-seq experiment. ChIP-seq detects the amount of specific DNA fragments directly and indirectly bound by the protein-antibody complexes. Mapping the extended DNA fragments on the genome, an appropriate clustering of overlapping fragments and filtering of the clusters result in an identification of putative TFBSs. Thus the integrative relative avidity of TFBS in ChIP-seq experiment could be quantified by the number of overlapping DNA fragments in the cluster loci. When this number is equal to or larger then a specificity threshold, t, such significant DNA loci (specific BEs) could be used to construct and analyze of statistically reliable part of the empirical frequency distribution representing relatively high-avidity binding loci of specific TF-DNA binding. In particular, we used this part of the distribution for estimation of the number of reliably detected specific TFBSs (called , see Definitions & Methods).

In the datasets [15], which we used in our study, the specificity threshold was calculated using three different methods. The first method implements a model of normalization of observed binding peak height values in a ChIP-seq experiment against peak height values in the corresponding negative control experiment (anti-GFP [15]). However, the control data provided many strong peaks which are often found in specific genomic regions like satellite repeats creating an unpredictable bias in identification of specificity threshold. We found also a fraction of loci in which negative control and experimental set binding signals (peaks heights) are highly correlated and the fraction of locus in which the peaks in control dataset were higher than in the experimental set.

After removing non-random false peaks, a relatively small reduction of the number of the nonspecific DNA fragments even among the smallest peaks (1, 2,..., 7) about 15-25% of total sequence read could be excluded. However, many millions DNA fragments within low- and moderate- abundant peaks of are present in the data sets (data not shown). These findings suggest that the number and the variation of DNA fragments of the negative control experiment do not allow us to explain the source(s) of the major fraction of non-specific DNA sequences and their clusters occurred in a specific ChIP-seq experiment.

The second method implements a model of random occurrence of DNA fragment clusters in the genome. The model assumes that each genomic position has the same probability of producing a ChIP-seq DNA fragment (sequence read) and the probability of finding by chance *m *DNA fragments in a ChIP-seq experiment within the same genomic region taken from a genome of size *g*. The probability calculations were made, using a Monte-Carlo simulation, by randomly extracting 200 bp DNA fragments from reference mouse genome (mm8) and estimating the expected numbers of non-specific overlap peaks with various height values. Then the frequency distribution of the number of random ChIP-seq DNA fragments in a cluster overlap (peak heights) was constructed and the threshold value at the given specificity level for the original empirical frequency distribution of the peak height value was defined. The binding loci reported in [15] has been limited to the locis with specificity threshold value defined by the model described above, which was considered as the random background noise mode [15].

**Figure 2:**
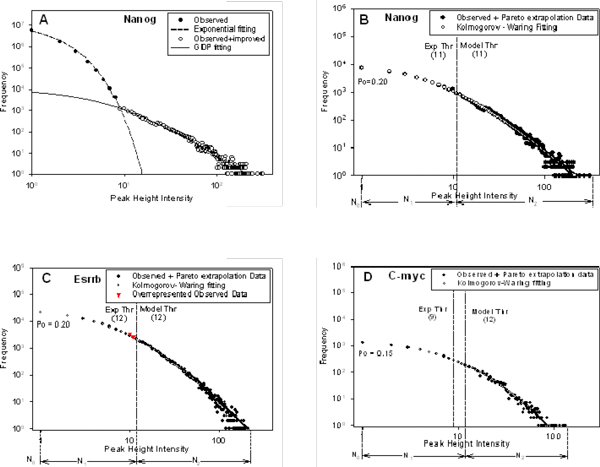
**Observed and predicted statistics of TF--DNA BEs**. A: Fitting and back extrapolation analysis for complete dataset. Decomposition of mixture model (1) for Nanog TF-DNA BEs is provided based on curve-fitting analysis of the model. Close circle: number of loci of ChIP-seq extended DNA cluster overlaps from 1 to 8 BEs. Open circle: number of loci of ChIP-seq extended DNA cluster overlaps from 9 to 73 (included) BEs. Noise-like (close circles) data fits well be exponential function with exponent parameter *s *= 1.05 ± 0.055 (p < 0.0001, t-test). The reliable set of TF BS (at >8 BEs) are equally well fitted by the left-side truncated GDP function (at *k *= 1.81 ± 0.15 (p < 0.001, t-test) and *b *= 8.00 ± 1.335 (p < 0.001, t-test)) as well as by K-W function (*θ *= 0.999, a = 6.618, b = 8.29; Table 3). Extrapolation curve predicts the number of Nanog TFBSs in the noise-enriched binding site fraction of the empirical distribution. B: Nanog TF-DNA BEs, C: Esrrb TF-DNA BEs and D: c-Myc TF-DNA BEs. B, C and D: K-W model fitting on the observed and extrapolated of double-truncated GDP data to calculate *p*_0_. Vertical dotted lines are representing qPCR-defined threshold and the threshold defined based on best-fit double-truncated GDP function. Triangle symbols show the observed over represented number of TFBSs in compare to best-fit GDP function. *N*_0_, *N*_1 _and *N*_2 _are the numbers of non-detected, potentially detected and high specific (reliable) TFBSs, respectively. More detail information about parameter values of GDP and K-W models presents in Additional File 3, 4, 5.

The third approach based on qPCR validation data with correction of threshold value for a limited number of ChIP-seq DNA loci has been used [15]. ChiP-seq data analyses showed that in all cases the simple model (with a random uniform distribution of DNA fragments locations in the genome) produces too optimistic specificity threshold values in comparison with the values defined by q-PCR method [15]. For example, for TF Nanog the model overlap peak value 7 is predicted as a cutoff at FDR<0.95 [15]. At this cut-off value 32773 putative ChIP-seq BSs were predicted as "true" (or specific) TFBS. However, the analysis of 37 ChIP-seq defined BSs using ChIP-qPCR suggested the specificity threshold value equal to 11 (at specificity level 98%). At this specificity threshold value, only ~30% (10343/32773) of DNA fragment clusters could belong to "true" BSs. Additionally, the numbers of singletons (single unique ChIP-seq DNA sequences occurring only once per a single unique locus) generated in the computational simulation were systematically larger than observed ones (for example, Figure 2A). Inversely, predicted numbers of random clusters with low- and moderate- avidity loci (e.g. with overlap peak heights 2-10) might be systematically underrepresented in ChIP-qPCR data.

ChIP-qPCR is often used to determine in vitro whether a given ChIP-seq DNA fragment cluster belongs to a specific TFBS and, subsequently, to estimate the specificity threshold for the original ChIP-seq dataset by extrapolation. However, due to the limited number of loci which could be used in ChIP-qPCR, a sub-optimal design of ChIP-qPCR experiment might contain a bias in estimating the ChIP-seq BE specificity threshold. In this section we shall give an answer to the question: how to optimize a design of ChIP-qPCR experiments to minimize the sample size bias?

**Figure 3:**
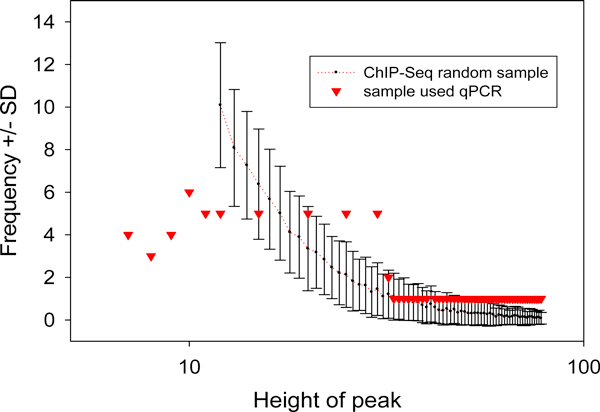
**Suboptimal design of the ChIP-qPCR experiment**. Statistics of BEs in Esrrb TF ChIP-seq data is following to skewed distribution. Difference in the frequency distributions of BEs for peaks used in qPCR and in random samples chosen from Esrrb TF ChIP-seq library at peak values >11 available for this dataset. In Chen et al [15], to determine the specificity, the peak height critical threshold were determined by 3-fold enriched qPCR signal/noise criteria.

On Figure 3 we present the results of our analysis of 94 ChIP-seq loci containing 224 distinct DNA fragments from Esrrb TF ChIP-seq library selected by the authors of [15] for their validation of computational model estimate (t = 6). Figure 3 shows that ChIP-qPCR experiment was designed on the studied affinity value range based on a uniform distribution function of the number of TF-DNA BEs per locus. To search for a more realistic frequency distribution representing Chip-seq data, 224 random samples of the 94 ChIP-seq loci were chosen without replacement from the same ChIP-seq library. On Figure 3 the mean values and standard deviations (SD) of the number of BEs (peak heights) over these 224 samples are plotted. This figure shows that the observed frequency distribution of mean peak height is a skewed Pareto-like function. In comparison to the uniform distribution, our frequency distribution is significantly overrepresented by the BS with overlap peak values less than 13 and is significantly underrepresented by the with overlap peak values greater than 15. This result suggests that a sub-optimal design of a qPCR validation study can provide a false positive increment in the estimation of the number of q-PCR-confirmed specific ChIP-seq loci and thus it can erroneously predict a larger specificity value than it can be deduced from the whole ChIP-seq dataset. If so, the actual specificity threshold values of ChiP-seq experiments might be larger than it was reported in [15]. Respectively, the real number of specific TFBSs at the threshold value given by qPCR test could be smaller than it is expected from results of the test. In the next sections we will present an alternative computational method which uses all available Chip-seq data and estimates the specificity of Chip-seq assay without (i) assumptions regarding physical distribution non-specific Chip-seq DNA clusters (noise BEs) and (ii) the need for a validation of the results in ChIP-q-PCR assay.

**Table 1:** Comparative analysis of TF binding specificity estimated based on three methods. Specificity threshold estimates by the uniform noise model [15], by ChIP -qPCR [15] and by the best-fit GDP function. The uniform random model-based estimations of the threshold values defined at FDR <5%. TF binding specificity threshold *t *was estimated based on the best-fit double truncated GDP function. The last two columns of the table shows that GDP-model could improve the specificity estimates providing by the Poisson model and ChIP-q-PCR assay.

TF	Unique mapped fragments, M*	Noise threshold by Poisson model*	# q-PCR experiments*	Noise 3-fold enrich threshold by qPCR*, q	Specificity threshold by GDP, t	**at t**	*N*_2 _at q*	**at q**	Specificity by qPCR, %	GDP-predicted specificity at q
Essrb	3609843	5	94	12	12	21646	21646	21600	94	99

Nanog	8424102	7	37	11	11	10343	10343	10213	97	98

oct4	4911144	6	47	8	11	1697	3761	2942	95	78

Sox2	4821446	6	48	8	13	2082	4526	5196	97	85

E2F1	8787961	6	48	9	16	13741	20699	21122	100	98

Tcfcp2I1	8449181	6	47	9	17	16293	26910	27912	97	96

ZFX	3844429	5	52	9	11	7161	10338	10966	100	94

Klf4	3807970	5	47	8	10	7433	10875	12122	97	88

c-Myc	6637404	7	48	9	12	1980	3422	2632	100	77

n-Myc	4823212	6	46	8	13	3214	7182	8780	97	78

STAT3	5351116	6	48	8	11	1229	2546	1983	97	78

### A mixed model of the sample-size dependent frequency distributions of binding events could be used to estimate the number of low/moderate- and large- avidity binding loci

A simple visual inspection of the available empirical frequency distributions of TF-DNA binding plot in log-log scale (Figure 2, Additional files 1, 2) allows us to suggest that a mixture of at least two distinct skewed sample-size - dependent frequency distributions of binding avidity is present in the data. Statistical analysis of ChIP-based [9,11-15,19] has showed that the left part of the empirical distribution is reach with the noise resulted from relatively low avidity BE and the right side of the distribution is represented by relatively high-avidity TF- DNA binding (Figure 2, Additional files 1, 2). Also we found that the tails of the empirical distributions of TF-DNA binding avidity exhibit monotonically-skewed shape with a greater abundance of low avidity and more gaps among the high-avidity loci. Low- and moderate- avidity TFBSs are highly-abundant in the mouse and other mammalian genomes and could play biologically meaningful functional roles.

The specificity threshold of the distribution of the number of TF-bound DNA loci, *t*, is the parameter which separates lowly reliable and highly reliable-binding loci at a given specificity level (6).

For quantitative analysis of ChIP-seq data based on the model (1) it should be important to estimate the specificity threshold, *t*, as well as the number of high-avidity specific loci , at avidity *m *≥ *t*, and the number of specific loci  having low/moderate avidity less than *t *(*m *<*t*). To do that, we use the *fitting and back-extrapolation *method (see Methods). We illustrate our method with the analysis of the ChIP-seq Nanog TF-DNA binding data as an example (Figure 2A &2B).

We demonstrated that the *exponential *distribution function fits well to the background noise of the empirical distribution of TF-DNA binding, while the *truncated GDP function *(2) fits well to long tail of the empirical distribution (Figure 2A). Specifically, this noise-like part of the distribution can be fitted well with an exponential function with exponent parameter *d *= 1.05 ± 0.055 (p < 0.0001, t-test) (Figure 2A). Table 2 and Additional File 3 (Table 1) provide the estimated parameter values and detailed characteristics of statistical tests.

**Table 2:** TGDP function parameters for the eleven TF libraries. Non-linear regression module of SigmaPlot program was used to estimate parameters of GDP function. More details regarding binding statistics and TGDP function parameters are presented in Definitions, Methods and Additional files.

TF	GDP-defined specificity threshold t	**at t**	J	*k *± *SE*	*β *± *SE*	# GDP-predicted seq.,	#Observed seq. at GDP specificity threshold t, *M*_2_	**/*M*_2_**
Esrrb	12	21646	338	2.40 ± 0.077	10.42 ± 0.682	570938	551406	1.04
Nanog	11	10343	312	1.81 ± 0.156	8.00 ± 1.33	297493	299830	0.99
Oct4	11	1697	195	3.00 ± 0.494	7.66 ± 2.910	34153	30637	1.11
sox2	13	2082	206	2.35 ± 0.225	3.03 ± 1.432	49976	44023	1.14
E2f1	16	13741	399	3.55 ± 0.006	46.24 ± 0.340	553688	517826	1.07
Tcfcp2I1	17	16293	382	1.50 ± 0.077	9.28 ± 1.227	771652	839103	0.92
Zfx	11	7161	197	2.90 ± 0.162	3.73 ± 0.778	129813	123943	1.05
Klf4	10	7433	184	4.83 ± 0.335	11.16 ± 1.421	112898	107891	1.05
c-Myc	12	1980	132	2.94 ± 0.742	16.24 ± 7.298	47030	44402	1.06
n-Myc	13	3214	119	2.81 ± 0.228	4.01 ± 1.306	68725	65302	1.05
STAT 3	11	1229	173	4.02 ± 0.650	16.19 ± 4.248	24209	22635	1.07

The most noise-affected data points are located at the left side of the empirical histogram (from 1 to 8 BEs), where the data was accurately fitted by the exponential function using Sigma-Plot software (Figure 2A). The values of the exponential function at data points from 0 to 9 were fitted (solid line on Figure 2A) and for larger values were estimated by the best-fit exponential function (extrapolating onto the right). At the specificity threshold (*t *= 11) identified from the analysis of ChiP-q-PCR data, the admixture of non-specific BS (originating from the tail of the exponential function) consists of ~2% of the reliable subset (Sp = 98%, respectively). Importantly, at the predicted specificity level (Sp = 97%) our best-fit mixture model predicts the same specificity threshold (*t *= 11).

Subtraction of the exponential function values from the observed frequency distribution values allowed us to restore the "noise-free" frequency distribution function for overlap peak value 9 and larger (Figure 2A &2B). This reconstructed double-truncated frequency distribution could be considered as a *reliable *segment of the empirical specific binding avidity distribution. After parameterisation of the GDP function we can extrapolate the best-fit GDP to smaller overlap peak values (8 and smaller). Figure 2A shows that the truncated GDP function fits well to the right-tail of the empirical frequency distribution of TF specific binding in the interval of peak values larger than 11. This part of the empirical distribution characterises the more reliable fraction of TF-DNA binding loci corresponding to high-avidity level BSs and represented in ChIP-seq experiment by number of overlapping DNA fragments (peak values) up to the maximal observed number of overlaps.

The GDP function, after parameterization in this interval, can be used to predict the values of the function for smaller number of peak values: 8,7,6,...,1. At the threshold 11 Sp= 97%, and the number of GDP-estimated specific BSs equals 10213 versus 10343 BSs observed in Nanog ChIP-Seq assay (Table 1). The number of observed unique ChIP-seq DNA fragments, *M*_2, _representing these BSs (*N*_2_), equals 299830; GDP estimates 297493 ChIP-seq DNA fragments. Interestingly, the last number represents only 3.5% of the total number of the unique DNA fragments of the ChIP-seq Nanog library. Similar results we obtained for other TF libraries (Table 1). These results suggest that the major fraction of ChIP-seq DNA fragments maps to moderate and low avidity loci specific TFBSs and to background non-specific DNA loci.

Only part of original ChIP-seq sequence datasets is available [15]. The analyzed BEs were limited in the dataset to the loci conforming to the specificity threshold value defined by the random model of uniform distribution of background signals reported in [15]. Using these data sets, the smallest threshold of the most reliable and the most specific BEs (providing the best goodness-of fit statistical criteria, according to [20]), was used to calculate the GDP-defined specificity cut-off value. In these cases, the right tail of the available truncated empirical distribution was used to estimate the parameters of the truncated GDP function.

We calculated the specificity threshold values (8) starting from ChIP-qPCR-defined specificity threshold followed by minimizing the distance between the truncated GDP probability function and the observed frequency distribution. Figure 2 and Additional Files 1, 2 show that the truncated GDP function fits well the right-tail GDP function corresponding to the fraction of reliable and highly specific BEs. Moreover, due to the high accuracy of parameterization of the mixture GDP function and the exponential function, our curve-fitting of ChIP-seq data allows us to predict the GDP function for the noise-enriched BEs, located on the histogram in the left part in the empirical avidity distribution function. Figure 2C and Additional File 1 show the truncated frequency distribution of Esrrb TF-DNA binding (overlap peak cut-off value 12) and the best-fit GDP function extrapolated to the noise-enriched part of the distribution are presented. The best fit GDP function with parameters *k *= 2.40 ± 0.0778, *b *= 10.42 ± 0.6828 allows us to extrapolate the best-fit curve and to predict the number of Esrrb TFBSs the in the noise-enriched binding sites.

Figure 2D contains the results of curve-fitting analysis for the c-Myc ChIP-seq library. It shows that the best-fit GDP function, by extrapolation, could provide an estimation of specific BEs in the highly noisy region of the distribution (the left part of the distribution. Thus, based on these findings, the empirical frequency distribution of TF BEs could be separated into the right-side and left-side regions, relatively to the critical cut-off value, *t*, discriminating the reliable and strongly-specific BSs from the less reliable noise-rich and low/moderate avidity BSs.

### The analysis of fitting the avidity curve with GDP could improve the specificity threshold obtained from ChIP-q-PCR

Figure 2D shows that at the given level of specificity (sp > 97%) the best-fit GDP function can predict a similar or larger binding specificity threshold, t, than the one obtained from ChIP-q-PCR data. Suboptimal design of the ChIP-qPCR experiment possibly (Figure 3) supports this suggestion. Table 1 and Additional file 2 show that in some Chip-seq libraries the frequency values at qPCR-defined specificity thresholds and the values around them systematically deviate from the GDP extrapolation curves. For six TFs (Oct4, sox2, Klf4, c-Myc, n-Myc, STAT3) the specificity predicted by our model at the qPCR-defined critical threshold values was much smaller (cutting off specificity at 78%, 85%, 88%, 77%, 78%, 78%, respectively) than it was found in qPCR-ChIP experiments (Table 1). In these cases our model allows us to select more reliable critical threshold values (at a larger overlap peak height) following a single confidence criterion (e.g. specificity cut-off at 94%). For the other four TF ChIP-seq libraries,(Essrb, E2F1, Tcfcp211, ZFX) out of 11 studied TFs specificity thresholds were defined by the both methods at a similar specificity cut-off (94%) (Table 1). In particular, for c-Myc BEs, at specificity level >0.95, reported in Supplementary File of [15], for qPCR-ChIP our model predicts *t *= 12 instead *t *= 9 (determined by qPCR-ChIP experiment with Sp = 100% based on 47 qPCR experiments). We could suggest that in these cases "optimistic" specificity estimates could be obtained due to a sub-optimal design of qPCR-ChIP experiments (see discussion of Figure 3) and/or unreliable detection of the peak values for a given range of overlap peak signal enriched by non-specific BSs.

### Estimation of the number of ChIP-seq DNA fragments in predicted specific low- and moderate- avidity binding loci

Parameter *a *of our mixture model (1) was estimated as the fraction of specific DNA fragments in the specific TF-DNA binding loci in the ChIP-seq experiment. The value of this parameter we calculated by extrapolation of the best-fit GDP function or the best fit K-W function to low- and moderate- values of peak heights on the empirical histogram of binding avidity. (7). (see Table 3). The estimated values of parameter a show that estimated specific DNA fragments is much smaller than a fraction of non specific background DNA fragments. This parameter varies significantly across the libraries. (table 2S of Additional file 4). We suggest that parameter *α *can be considered as an important parameter characterising an enrichment of the library with sDNA fragments bound to the specific lociBEs. These parameters can serve as targets for further optimization of ChIP-based TF-DNA biding assays.

**Table 3:** Best fit K-W function estimations: specific components of probabilistic TF-DNA binding model

TF	*θ*	*a*	*b*		*α*		*p*_*o*_		*N*_2_	*Se*, %	*N*_2_/ * 100%
Esrrb	1	13.1	15.87	916615	0.25	114171	0.2	142713	21646	80	15

Nanog	0.999	6.618	8.292	414084	0.05	40891	0.2	51114	10343	80	20

Oct4	0.988	5.681	8.32	84810	0.02	19160	0.32	28176	3761	68	13

Sox2	0.99	1.844	4.023	208548	0.04	67633	0.54	147028	4526	46	3

E2f1	0.99	34.569	36.837	668875	0.08	37302	0.06	39682	20699	94	52

Tcfcp2I1	1	7.817	9.198	1118336	0.13	67894	0.15	79875	26910	85	34

Zfx	0.985	2.224	4.833	655223	0.17	236320	0.54	513739	10338	46	2

Klf4	0.985	8.055	12.26	416960	0.11	117698	0.34	178330	10875	66	6

c-Myc	0.999	13.1	16.35	70735	0.01	8393	0.15	9874	3422	85	35

n-Myc	0.988	2.6	5.11	365150	0.08	120891	0.5	241782	7182	50	3

STAT 3	0.99	12.931	16.408	48016	0.01	8177	0.21	10350	2546	79	25

### Fitting-Extrapolation method for K-W function and estimation of parameters *p*_0 _and *N*_*tot*_

In this section we will answer the question: how many physically specific BSs of a given TF exist in the mammalian genome? We used the theoretical result obtained in the previous section to estimate the parameters *a*, *b *and *θ *of K-W function and thus, to estimate *p*_0_. Practically, for all ChIP-seq datasets we could fit the K-W probability function to the GDP function values after fitting the truncated GDP to a high-confidence segment of the empirical frequency distribution of TF-DNA binding. This segment of the empirical distribution is assigned by the number of ChIP-seq DNA fragments, called *M*_2_, which form *N*_2 _clusters. The number of these clusters was counted for the peak height values ranged between specificity threshold level *t *and maximum value of peak height *J*, where the specificity *Sp *is defined by the formula (8).

The detailed description of the algorithm of estimation of the parameters of skewed distribution function functions like GDP and K-W was reported in [20]. Using this algorithm, we found that for ChIP-seq data the GDP function exhibits a fairly accurate approximation of K-W function throughout the entire range of BEs (Additional file 5), We used the properties for fitting K-W function based on the extrapolated data estimated from the best-fit truncated GDP function values. The estimated parameters of K-W function, we used to estimate the probability of non-detected BEs (*p*_0 _at *m *= 0) by the formula (28) and *N*_*tot*_, as follows:

and estimate the number non-observed TFBSs

Using *p*_0 _(28)_, _we could estimate TF-DNA binding sensitivity

### Kolmogorov-Waring Function predicts a large number of TFBSs with low- and moderate- binding avidity

GDP can be considered as a good empirical approximation of the K-W function (see Methods). Granted this property, we used fitting and back-extrapolation method to get an accurate estimate of three parameters of K-W function. Figure 2C-D Table 3 shows that all empirical distributions of binding avidity are fitted well by the K-W function. Using the estimated parameters of K-W function (see Methods), we can predict the fraction of specific BSs which have not been detected in the ChIP-seq experiments (*p*_*o*_) (28). For example, the parameters of the probabilistic model for Nanog TF data were estimated as *θ *= 0.997; *α *= 5.870; *β *= 7.465, and thus, from (28), we have *p*_0 _= 0.22. For Oct4 data: *θ *= 0.998; *α *= 5.681; *β *= 8.32, thus by (28) we have *p*_*o *_= 0.32. Interestingly, for all TF binding data the parameter  of the K-W function equals to or slightly smaller than 1. This result suggests that for TF-DNA binding-dissociation processes the rate of preferential dissociation equals to or little bit larger than the rate of preferential binding.

Using our parameterization algorithm we can estimate the total number of specific BSs in the mouse genome for a given TF. This estimate equals ~6.67 10^4 ^BSs for Nanog and ~2.82 10^4 ^BSs for Oct4. Table 3 shows the estimates for other TFs.

**Figure 4:**
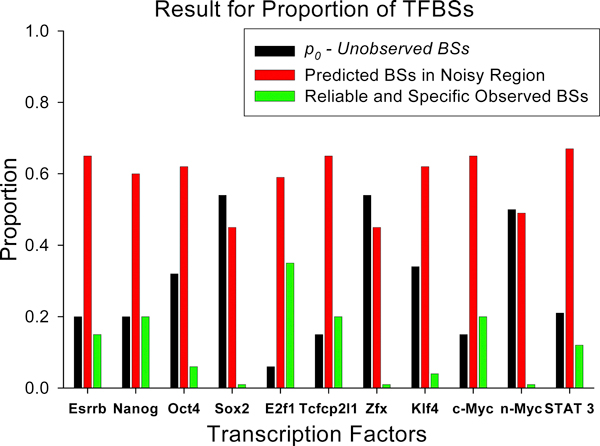
**Three segments in the range of TF-DNA BEs count for 11 TFs of mouse E14 embryonic cells**.

Reliable data points in the right-tail region of the empirical frequency distribution (starting from our best-fit GDP-defined cut-off peak value; region *N*_2 _on Figure 2B, C, D) and the best-fit predicted GDP data points of the noise-rich (left size) region of the distribution (region *N*_1_, Figure 2B, Additional file 2) were combined together. K-W function fitting was used to estimate the fraction of non-detected BEs, *p*_0_, and the total number of TFBSs, *N*_*tot *_(*N*_*tot*_*= N*_0_*+ N*_1 _*+ N*_2_). For example, for the Nanog dataset, at threshold 11, 1.02*10^4 ^reliable BSs were predicted by the GDP and K-W. Additionally, K-W function predicts in total 51114 Nanog BSs in the genome of E14 cells, where 10223 (20% of *N*_*tot*_) were non-observed BSs (*N*_0_) in the library; 30678 (60% of *N*_*tot*_) specific BSs where predicted in the noise-rich BE set (*N*_1_) and 10213 (20% of *N*_*tot*_) were predicted in the reliable specific BE set (*N*_2_), respectively (Figure 4). The estimates of the parameters of GDP and K-W functions and *N*_*tot *_for c-Myc, Esrrb and other TF data are given in Table 3 and, partially, in Figure 4.

Figure 4 and Table 3 shows that the fraction of non-detected BSs (*p*_0_) varies among different TF ChIP-seq libraries from 6% (E2F1) to 54% (ZfX) of the total number of BSs predicted in the genome of mouse ESC E14. The total number of predicted BS ranges from 9874 (c-Myc) and 10320 (STAT3) to 178330 (Klf4) and 513739 (ZfX). The total number of c-Myc TFBS predicted in the genome mouse ESC is similar to the predicted number of c-Myc TFBS predicted in human B-lymphocytes [12]. Note, later estimate derived based on the data analysis and modelling ChIP-PET data. Figure 4 shows also that reliable and specific BEs form the smallest fraction of the BSs with one exception (E2F1, 52%; Table 3), while the number of low- and moderate- avidity TFBSs for other ten TFs contain the most abundant subsets of *N*_*tot*_. The non-observed fraction of TF BS is estimated as the largest for Sox2, ZfX and n-Myc. Thus, these results strongly suggest that the sensitivity of ChIP-Seq technique is still low and has to be improved essentially.

### Significant number of putative low- and moderate- avidity TF binding loci are admixed to the noise-rich binding loci and might be functionally important

**Figure 5:**
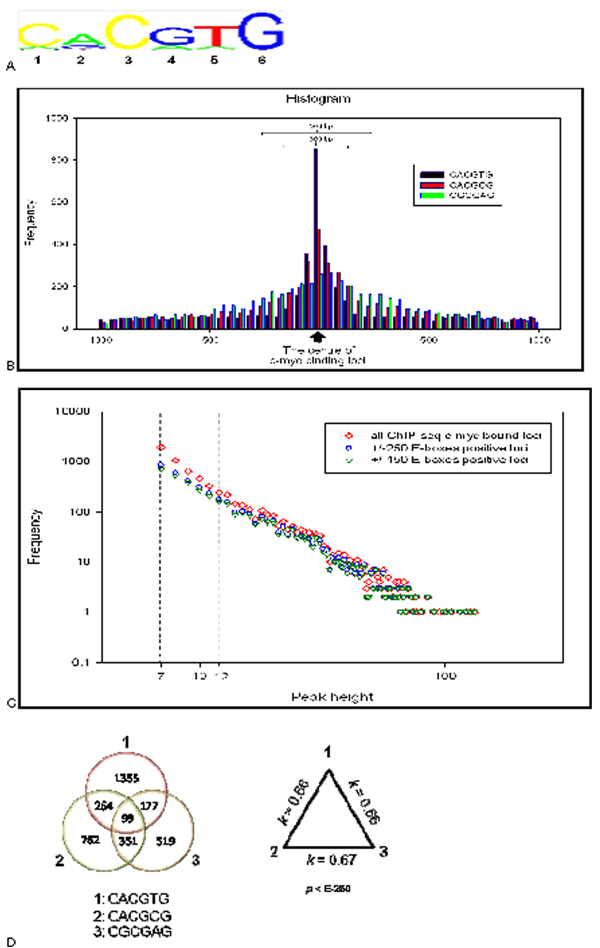
**Validation of ChIP-seq defined c-Myc binding loci based on motif finding analysis**. A: PWM of c-Myc TFBSs defined with NestedMICA program trained with 12 peak height or higher value defined in ChIP-seq experiment. B: Distribution of E-box sequences in ± 1 kb from the centre of ChIP-seq defined binding loci. C: Frequency distribution of the number of ChIP-seq overlapped DNA fragments (peak height). ◊: All ChIP-seq c-Myc bound loci for observed peak heights. o: E-boxes positive loci found in vicinity ± 250 bp. ∇: E-boxes positive loci found in vicinity ± 150 bp. D: Venn diagram of co-occurrence of E-boxes in ± 150 bp of c-Myc binding loci (left side). Pair of Kappa correlation coefficient of co-occurrence of E-boxes in c-Myc binding loci (right side).

According to the prediction of the GDP and K-W models, the number of low- and moderate- avidity TFBSs should monotonously increase when the number of unique overlapping ChIP-seq DNA fragments in a binding locus becomes smaller. To validate this prediction, we used motif-discovery program nminfer (see Methods section) to identify c-Myc binding motifs/E-boxes in the ChIP-seq binding loci. As a training set we used the loci with c-Myc-DNA binding specificity cut-off values defined by GDP model at ≥12 sequences per locus representing high avidity loci. We found Position Weight Matrix (PWMs) which contains 'canonical' E-box CACGTG 'non-canonical' E-boxes including CACGCG, CGCGAG and CACATG [18] and a novel non-canonical E-box CGCGAG which is found in our study (Figure 5A). All four non-canonical motifs were scanned for exact matches in the mouse genome (mm8) using SeqMap software [21]. The total numbers of matches of CACGTG, CACGCG, CGCGAG and CACATG in the mouse genome were 262,133, 83,490, 41,394 and 2,451,550, respectively. E-box CACATG sequence is highly frequently occurred in non-genic low-complexity regions of the mouse genome associated with repeats elements or promiscuous genome regions. To minimize false-positive or bias in the results, we excluded the E-box CACATG from our validation and prediction analyses. Then we studied the localization of the E-boxes CACGTG, CACGCG, CGCGAG within ChIP-seq binding loci. To do that we construct the frequency distribution of the E-box sequences around the central nucleotide of ChIP-seq-defined c-Myc biding loci (Figure 5B). To identify the region of the E-box localization within binding loci, we narrowed the scan region with ± 150 bp (300 bp) and ± 250 bp (500 bp) around the center of c-Myc binding locus (Figure 5B).

In total, 6437 ChIP-seq c-Myc binding loci with the peak values 7 and higher were found. We found that the number of ChIP-seq loci containing the E-boxes in ± 150 bp region is 3527 (Additional files 6A and 7A) and in ± 250 bp region is 3948 (Additional files 6B and 7B), respectively. These results suggest that c-Myc binding loci are strongly enriched with E-box sequences: we found that 55% (3527/6437) loci of the ± 150 bp region (Additional file 7A) and 61% (3948/6437) loci of ± 250 bp region (additional file 7B) are E-box-positive. Each of these regions exhibits at least one copy of the three specific c-Myc E-boxes or (CACGTG, CACGCG and CGCGAG).

Figure 5C shows the frequency distribution of the peak height representing c-Myc-DNA binding avidity starting with peak value 7. On the Y-axis, we present the numbers of ChIP-seq c-Myc binding loci at the given peak height value, the number of ChiP-seq binding loci containing E box in the ± 150 bp region of centre of locus, and the number of Chip-seq binding loci containing E-boxes in the ± 150 bp region of centre of locus.

Each frequency distribution on Figure 5C shows a similar trend to increase number of binding loci when the peak height decreases. For example in ± 150 bp regions (Additional file 7A), the number of the binding loci containing E-boxes with overlap peak values 7, 8, 9, 10, 11 and 12 were 721, 539, 378, 266, 209, and 159, respectively. The low- and moderate- avidity BSs with peak values from 7 to 11 include the major fraction (~60% (2113/3527)) of the binding loci containing E-boxes found in the ChIP-seq library. We also observed that the number of binding loci containing only one of three E-box sequences increases when the binding avidity decreases (not presented). These trends are in agreement with predictions of our model of TFBS binding (Figure 2, Additional Files 1, 2).

It has been shown that relatively high avidity TFBSs could be preferentially located nearby transcription start site (TSS) of the target genes and overlap with CpG Island [15]. It is also true for a large number of low- and moderate- avidity c-Myc BSs in the genome of human B-cells [12]. We observed that for mouse EC c-Myc ChIP-seq data [15], E-box-positive Chip-seq binding loci are also often localized in the putative promoter regions of target genes. We found that 50.9% (3277/6437) c-Myc binding loci with peak height 7 and larger are localized within ± 1 kb TSS region of 3966 RefSeq genes (mm8) (Additional file 8). We count also the number of c-Myc binding loci containing E-boxes and being around ± 1 kb of TSS. 68% (2223/3277) of c-Myc binding loci around ± 1 kb of TSS contain at least one E-box in ± 150 bp around the centre of c-Myc binding loci while 32% (1054/3277) of c-Myc binding loci have no E-boxes (Table 1 in Additional file 8A). When we extend the region from ± 150 bp to be ± 250 bp around the centre of c-Myc binding loci, percent of c-Myc binding loci around ± 1 kb of TSS contain at least one E-box increase to 76% (2493/3277) and percent of c-Myc binding loci without E-boxes leave 24%(784/3277). These results easily demonstrate strong association of the E-box-positive binding loci with gene target promoter regions. Perhaps, the most of c-Myc target genes in mouse EC cell genome should be considered as direct gene targets, because alternative E-boxes (for instance, CACATG) also found in the c-Myc binding loci with high frequency.

Interestingly, 28-30% of c-Myc binding loci containing at least one E-box sequence exhibit low- or moderate- avidity (peak height 7-8) c-Myc binding (Table B Additional file 8B). These results support the prediction of our probabilistic model (K-W) that, in low- or moderate- avidity binding could be considered as true c-Myc binding sites. However information about conserved motifs and other regulatory regions are required for indentifying putative true binding sites in low- or moderate- avidity of binding. By our analysis, E-boxes positive binding loci often overlapped with CpG island region(s) which are co-occurred with c-Myc BSs [12]. The loci with peak height >8 are overlapped with CpG Island in 82% (1305/1598) cases (+/-150 region in Table B in Additional file 8); E-box-positive relatively low- and moderate- binding avidity (7-8 peak heights) loci are also overlapped with CpG Islands of the putative gene targets in 75% (469/625) cases. The genes having this moderate avidity BS might be considered as strong candidates for further bioinformatics analysis and experimental validation.

### c-Myc Binding loci could be represented by multiple copies of E-box sequences

We found that binding loci could be represented by multiple copies of E-box sequences: 3527 binding loci of ± 150 bp regions are represented by 5546 E-box sequences (mean. 1.57 (5546/3527) E-boxes per locus) (Additional file 9A), and 3948 binding loci of ± 250 bp regions are represented by 7182 E-box sequences (mean 1.82 (7182/3948) E-boxes per locus) (Additional file 9B).

Figure 5D shows that the E-boxes often co localized in the ChIP-seq c-Myc binding loci. For instance, Venn diagram on left panel of Figure 5D demonstrates that in ± 150 bp around a centre of binding locus 19% of E-box CACGTG sequence (363/1895) and 25% (363/1476) of E box CACGCG sequence are co -localized in the same binding locus. Kappa correlation coefficient, which measures co-occurrence of the events (StatXact 5; Cytel Software Co), equals 0.66 (p < E-230). Similar values of the correlation coefficient we found between two other E-box pairs, which are presented on the right panel of Figure 5D. Similar results we obtained when 250-nt vicinity around a centre of c-Myc binding locus was analyzed (not presented).

### A case-study of multiple occurrence of c-Myc E-boxes in ChIP-seq –defined promoter regions of embryonic SC-related genes

It was shown that the TFBS found in ChIP-seq and ChIP-PET study of c-Myc have a potential to stimulate embryonic SC-specific gene expression [12,15]. In this section, we shall consider the examples of association between binding avidity level and potential relation of nearby genes with stem-cell specific activity. 

**Figure 6:**
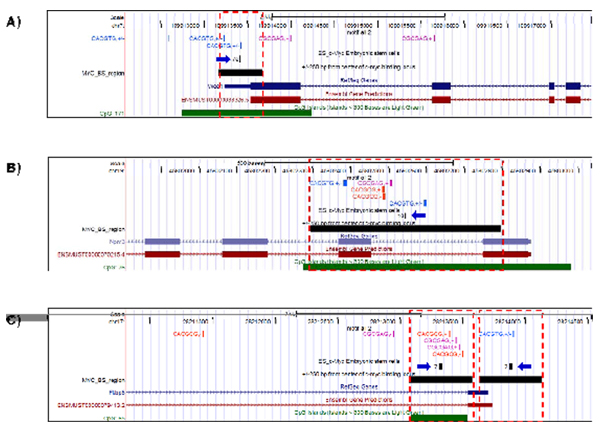
**Multiple occurrence of c-Myc E-boxes in promoter region around transcription start site (TSS) of c-Myc target genes identified in ChIP-seq experiment**. A: WEE 1 homolog 1(Wee1). The high-avidity (height peak = 76) c-Myc binding sites (pointed by blue arrow) in strong promoter region of Wee1 gene is supported with two canonical E-box CACGTG. The binding locus and E-boxes are overlapped with CpG Island which might be bound by c-Myc. B: Nucleoplasmin 3 (Npm3). The moderate-avidity (height peak = 10) as in the previous case the ChIP-seq c-Myc binding locus is located in the first intron promoter region. The locus is supported with five E-boxes: one canonical and three non-canonical E-boxes in first intron and another canonical E-box in second exon. In addition, this binding region and the E-boxes are located in CpG Island. C: FK506 binding protein 5 (Fkbp5). Two relatively low avidity c-Myc binding sites identified in ChIP-seq experiment confirmed with E-boxes. First ChIP-seq loci in upstream gene region has relatively low avidity biding site (height peak = 7) supported with canonical E-box CACGTG. The second ChIP-seq loci located in first intron and it is also relatively low avidity peak (height peak = 7) which is supported with two non-canonical E-boxes CACGCG and two non-canonical E-boxes CGCGAG. The last locus overlaps with CpG Island which suggests that this locus might be functional.

Figure 6A presents a genomic map of c-Myc E-boxes distribution in the WEE1 homolog 1 (Wee1) gene region. ChIP-seq c-Myc binding locus (pointed by blue arrow) should be considered as high-avidity TFBS due to peak height = 76. This binding locus contains E-box sequences of two canonical E-box CACGTG presented on Figure 6A. Wee1 was reported to be an important component of hESC cell cycle regulation pathway [22] and might be co-regulated with c-Myc [23]. In Figure 6B, a genomic region of Nucleoplasmin 3 (Npm3), a chromatin re-modelling protein responsible for the unique chromatin structure and replicative capacity of ES cells [24], is presented. A BS with moderate-avidity (height peak = 10), of c-Myc is located in the region nearby the first intron. The locus contains five E-box sequences: one canonical and three non-canonical E-boxes in the first intron and another canonical E-box in the second exon. In addition, this BS and the E-boxes overlap with a CpG island. Figure 6C shows c-Myc low avidity BS within FKBP5 (FK506 binding protein 5) promoter region. Two relatively low avidity c-Myc BSs contain c-Myc E-boxes. First BS located in the upstream region of the gene has a relatively low avidity (overlap peak value 7) and contains a canonical E-box CACGTG. The second BS located in the first intron also has relatively low avidity (overlap peak value 7) and contains two non-canonical E-boxes CACGCG and two non-canonical E-boxes CGCGAG. The last BS overlaps with a CpG Island which suggests that this locus might be related to the regulation of the transcription of the gene.

FK506 binding protein 5 (FKBP5) belongs to the family of immuneglobulins named for their ability to bind immunosuppressive drugs, also known as peptidyl-prolyl cis-trans isomerases, and also with chaperones (involved in protein folding) [25]. FK506 also plays an important role in cancer tumors growth and chemoresistance through regulating signal transduction of the NF-kappaB pathway [25]. The expression and activity of NF-kappaB is comparatively low in undifferentiated embryonic cells ES cells, but increases during differentiation of the ES cells [26]. Due to this finding we could suggest that c-Myc binds to the low avidity BSs in the promoter region of FKBP5 and, through a modification of expression level of this gene, can provide a regulation of NF-kappaB pathway in mouse ES cell. Interestingly, high, moderate, and relatively low avidity c-Myc BSs in the promoter regions of Wee1, Npm3, and Fkbp5 genes have their binding association scores correlating with ChIP-seq c-Myc binding avidity. Binding association score estimates the genomic distance between a BS and a gene TSS [15]. Additional file 10 contains the data demonstrating that Wee1 could be under promoter regulating activity of Oct4, c-Myc, and n-Myc, while Npm3 could be under promoter activity of c-Myc only. However, although Fkbp5 shows a moderate binding association score with c-Myc and STAT3, Fkbp5 expression is strongly associated with Nanog and n-Myc promoter activity. These results suggest that all three genes could be under the transcriptional control of c-Myc and, additionally, that Wee1 and Fkbp5 could be associated with ES-cell specific expression.

## Discussion

In this work, we studied statistical characteristics of protein-DNA binding events for the eleven stem cell- related transcription factors bound in the genome of mouse embryonic stem cells E14 and detected by ChIP-seq assay [15]. Several methods for ChIP-seq data analysis have been recently developed [11,13,27]. However, an appropriate mathematical model of TF-DNA binding in ChIP-seq binding assay and statistical evaluation of the sensitivity of the methods has been not developed. Several approaches to quantitative identification of individual TF-bound loci have been recently developed(peak finder algorithms [7,9,11-13,27]), however overall binding events for specific DNA loci including low- and moderate- avidity TFBS at the genome scale have been out of systematic consideration.

Our previous analysis of different ChIP-base TF-DNA binding datasets [9] suggested that the mixture probability distribution model (1) could reflect a common property of TF-DNA binding events in different cells in different experimental conditions. This model allowed us to estimate the specificity of ChIP-based TF-DNA binding events for several TFs. In the present study, we develop a mathematical model of TF-DNA binding-dissociation events in both TF-specific and TF-in specific genomic loci and use this model to estimate a number of essential parameters of statistical distributions observed in ChIP-seq assays. We include in our consideration the binding events of TFs in the entire range of their avidity to their binding loci from very high to very low. We focused on several practically important parameters closely related to analysis of the empirical TF-DNA binding distributions: *t*, *Se*, *Sp*, *p*_0_, and *N*_*tot *_(Figure 3, Table 3). Interestingly, for all TF binding data, the parameter  of the K-W function equals to or slightly smaller than 1. It suggests that the Waring probability function could be used as good approximation of K-W function for analysis of empirical frequency distributions of ChIP-seq binding. Another interesting finding: for TF-DNA binding-dissociation process in the mouse genome the rate of preferential dissociation equals to or little bit larger than the rate of preferential binding.

We found that the parameters of the mixture distribution are sensitive to 1) the sample size (number of non-redundant sequence tag reads) *M*, 2) the proportion of nonspecific sequence reads in the sample, 3) the sampling model and 4) the analytical models and the computational algorithm used for the parameterization of the distribution of TF-DNA BEs. We have demonstrated that skewed shape and sample size-dependence is a common property of a specific TF-DNA binding distributions [7,9,11,12]. Similar results we obtain here for 11 ChIP-seq samples derive from mouse embryonic SCs. Strongly positive values of parameter *β *in the GDP model describing TF-DNA binding in specific loci were found in all TF libraries (Table 2, Additional file 2 (Figure 2S)). Parameter *J *of the truncated GDP function reflects the maximal observed number of BEs of the GDP model. This parameter positively correlates with the sample size *M *[9,28]. These results agree with previous findings in ChIP-PET TF-DNA binding assay [9,12,13].

The values of observed and computationally predicted parameters of the statistical distribution k are found very close to each other for all ChIP-seq TF libraries (Table 2, Additional File 1, 2, 3, 4, 5). Using 11 available ChIP-seq TF-DNA binding datasets, we revisited and improved the estimates of the levels of sensitivity and specificity of the TF-DNA BEs.

Our mixture probabilistic model allows us to estimate the specificity cut-off value for ChIP-seq library and also estimates the fraction of specific TF-DNA binding loci for TFs in a ChIP-seq data. Our probabilistic model estimates also the specificity threshold, which value often is close or more stronger than estimates by ChIP-qPCR assay. Table 2 shows that for all 11 library our model (1) models provides >94% specificity. Thus, we conclude that the basic concept of mixture skewed scale-dependent distribution, originally developed for ChIP-PET data analysis [9,12], can be applicable to ChIP-seq data.

We conclude that a model of random occurrence of DNA fragment clusters in the genome is not appropriate for quantitative determination of critical threshold of binding specificity in Chip-based genome-wide binding analyses, including ChIp-seq. This conclusion is in agreement with recent computational simulations of the frequency distributions of TF-DNA BEs in Chip-Seq data [13], where local background noise for Stat1 TF ChIP-seq data [5] was modelled.

We developed a simulation model of unbiased identification of specificity of 'problematic' ChIP-seq loci. The method could be used to optimize the experimental design of ChIP-q-PCR experiments.

Experimental data, as it is demonstrated in this work, has noise- enriched BEs accumulated mainly below the specificity threshold values (Figure 2, Additional File 2).

Our probabilistic model shows that at the conventional specificity threshold % (> 95%), the fraction of high-avidity specific sequences and TF binding loci containing these sequences are surprisingly low in all studied libraries. These results suggest that the major fraction of true binding sites could not be detected by the ChIP-seq method without additional experimental validation and rigorous bioinformatics and extensive statistical analysis of data.

According to our model prediction, the number of low- and moderate- avidity TFBSs should monotonously increase when the number of unique overlapping ChIP-seq DNA fragments in a binding locus becomes smaller. To validate these predictions, we used motif-discovery program nminfer to identify a position weighted matrix (PWM) of c-Myc motifs in the ChIP-seq binding loci. Our bioinformatics and statistical analyses revealed that the moderate avidity BSs with peak values 7 to 11 include the major fraction (~60% (2113/3527)) of the E-box-containing TFBSs found in the ChIP-seq library. We also observed that the number of BSs, containing each of the three major c-Myc E-box sequences and their combinations, monotonously increases when the binding avidity decreases (Figure 5C). All these trends are in agreement with predictions of our model of TFBS binding (Figure 2, Additional Files 1, 2). Thus, in combination with motif-finding techniques (and/or experimental validation assay ChIP-qPCR) our modelling approach allowed us to identify the loci of many thousands of novel BSs with characterized with low- and moderate- avidity of TF. Moreover, the number of undetected low- and moderate- avidity specific TFBSs was estimated, which addresses common problem of sensitivity of a given ChIP-seq assay for a given TF in cells under given experimental conditions. We show that although ChIP-seq is a powerful technique still it produces essentially incomplete information about the low- and moderate- avidity TF- DNA binding events in the complex (e.g. mammalian) genomes.

Our mathematical modelling of the mixture strong and weak TF-DNA binding and sequence analysis of genome-wide binding data suggests that integration of these approaches could help to reveal many new target genes for c-Myc and for other studied TFs. For instance, best-fit K-W function predicts in total 51114 Nanog BSs in the genome of E14 cells: 20% of these 51114 Nanog BSs were non-observed in the ChIP-seq library and 60% of the BSs were predicted by the model in the noise-rich BE set. These model-based and data-driven predictions of Nanog BSs could be validated in case study experiments. A functional significance of such low- and moderate- avidity BSs for putative target genes might be investigated in a near future.  

The most important thing that our results suggest that low- and moderate- affinity BSs could have biologically meaningful functional roles. However, biological role of the enormous number of the moderate- and low- avidity BSs for TF is unknown. We speculate that these bindings could be used in the nucleus to storage large number diverse TFs and their cofactors at quasi-stationary and thermodynamically-defined states at vicinity of double-stranded DNA. Such weak, unstable and multiple protein-DNA bindings might be use by a cell for recruitment and redistribution of specific TF molecules along the double strand DNA depending from external and internal regulatory signals. We also speculate that the strength of TF-DNA binding-dissociation could be significantly modulated by cooperative interactions among TFs and DNA-binding signals.

For all studied TF library a relatively large fraction of low to moderate binding loci was not detected at all (Table 3). So the estimation of *p*_0_, by K-W model, is an informative measure of the incompleteness of experimental data. ChiP-Seq- derived frequency of DNA-TF BEs can be fitted well with truncated K-W probability function and thus, allows us to estimate all specific TFBSs (*Ns*_1_+*Ns*_2_) which could be found in the library and also to estimate the total number of TFBSs (*N*_*tot*_) entire genome.

However, father analysis of sensitivity and robustness of estimates and extrapolations of the probabilistic model of TF-DNA binding model and analysis more complete and large ChIP-seq datasets might be important for robust and accurate parameterization of our models and its applications.

## Conclusion

We proposed a probabilistic model of TF-DNA binding process at the genome level and based on the model we developed a computational method which allows us to 'de-noise' ChIP-seq datasets and to estimate the specificity and the sensitivity of ChIP-seq assays.

Goodness-of fit analysis of GDP and K-W functions suggests that the sensitivity problem has not yet been technically resolved by the ChIP-based methods, including ChIP-seq. TFBS motif finding analysis supports our results. Due to the proposed improvements in the sensitivity and the specificity of ChIP-seq assay, functional roles of an extremely large number of low/moderate avidity TF binding loci in the mammalian genomes can now be investigated. After these studies the models of transcriptional regulatory network in embryonic cells and other cell types should be carefully revised.

The numbers of the low/moderate- and high- avidity specific TF BSs are estimated here for all the studied data sets. We suggest that many low- and moderate- avidity BSs have biologically meaningful functional roles. Since in the previous studies only high avidity TF BSs could be reliably detected by ChIP-seq assays, identification of other binding sites and elucidation their functional role in genome is a great imperative goal for biotechnology, computational biology and functional genomics.

It is likely that our approach could also be applied to the analysis of high-resolution ChIP-based generated profiles of chromatin chemical modifications [29,30] in mammalian genomes. Our work provides a theoretical framework for a comprehensive computational prediction and a robust experimental identification of TFBSs (and other ChIP-seq data) when low- and moderate- avidity sequences are over-represented in ChIP-derived sequence samples.

## Methods

### ChIP-seq data sets

We used ChIP-seq datasets (ChIP-seq libraries) generated by Solexa sequencing for eleven TFs (Nanog, Oct4, sox2, KLf4, STAT3, E2F1, Tcfcp211, ZFX, n-Myc, c-Myc and Essrb). These TFs are considered as essential TFs for the maintenance of the pluripotency in mammalian stem cells and were studied using murine E14 embryonic stem cells cultured under feeder-free conditions as described in Chen et al [15]. The mapped ChIP-seq datasets were downloaded from T2G GIS DB (http://t2g.bii.a-star.edu.sg; see also NIH GEO ID:GSE 11431).The extended ChIP-seq DNA fragments were clustered and the number of overlapping fragments were summed at each locus and used to construct empirical frequency distribution of TF-DNA binding. Some statistical additional characteristics of the libraries are presented in Table 1 and Table 2 and Additional files 4, 5.

### Motif finding and target gene counting

To validate predictions of our TF-DNA binding model and to extend the sensitivity of ChIP-seq derived sequences clustered the loci with low- and moderate- binding avidity, we used genome coordinates of the available extended ChIP-seq fragments and provided computational identification of TFBSs by using motif-discovery program *nminfer *from NestedMICA http://www.sanger.ac.uk/Software/analysis/nmica/[31]. c-Myc TF ChIP-seq data [15] were used to illustrate our approach NestedMICA program was used to identify position weighted matrixes (PWMs) E-boxes and consensus sequence motifs of c-Myc TF into genome-mapped ChIP-seq DNA fragments. The sequences of strongly specific BEs with height 12 and higher (peak12+) were used as a training set for motif discovery. The training set sequences were downloaded from mouse genome (UCSC mm8 after repeat masked by capital Ns). The test set is peak regions (± 200 bp around centre of the cluster peaks) with height 7 and higher (peak7+), which have been not used in the training set.

The PWM found from training set were computationally scanned on the test set by *nmscan *program at score threshold-3.0. The motifs found from in test set were used to scan and count number of each motif found in the mouse genome (mm8).

### Empirical frequency distribution of the avidity of TF-DNA binding

To quantify the avidity of TF-DNA binding in a given locus, Chen et al. [15] extended the distinct fragments by 200 bps and clustered these fragments that were overlapping by at least 4 bps. In ChIP-seq experiment, "a binding signal" for a given binding locus has been represented the number of the DNA fragments formed by these 5-end "extended and overlapped" DNA fragments [15]. For Chip-seq experiment, this number could the considered as the TF-DNA binding event (BE) representing TF-DNA "binding avidity" averaged across genome BSs of hundred millions cells.

We define a list of uniquely mapped ChIP-seq DNA sequences observed in a given ChIP-seq experiment as the Chip-seq library. To quantify the data of an individual ChIP-seq library, we defined the size of the library, *M*, as the total number of distinct ChIP-seq reads uniquely mapped onto a reference genome. Let *m *denote the number of the BEs counted by a peak-finding program (e.g. T2G) in a DNA fragment cluster overlap of ChIP-seq library.  The single DNA fragment mapped on the genome is also included. *m *= 1, 2, 3,..., *m*_max_, where *m*_max_ = *J* denotes the maximum value of m. Let *n*(*m*, M) denote the average number of genome loci in which the BEs found in a given ChIP-seq data exactly *m *times in the library of a size *M*. Due to sample size, experimental errors and biological variation across  many cells and environmental conditions, observed *n* is the function averaged across the cells and conditions and this function increases when M becomes larger. Thus, *n *only approximates a true number of TFs bound to genomic loci with BE value *m*. However, *n*=*n*(*m*, M) after an appropriate normalization could be used for statistical analysis of relative binding avidity of ChIP-seq binding loci.

Let denote  the total number of distinct loci counted in the ChIP-seq library. Then  and we could call *M *also as the "DNA sequence mass". The empirical frequency distribution of the number of DNA fragments in the locus within the ChIP-seq dataset () might be considered as the empirical probability function of TF-DNA binding avidity. Such histogram is an essential starting point for further statistical analysis of data and planning of validations studies [7,9,13].

### An empirical probabilistic model

We assume that the probability distribution function of TF-DNA binding avidity in ChIP-seq experiments could be modeled as a mixture of two probability distribution functions(1)

where *P*_*s *_is the probability distribution function of specific binding *P*_*ns *_is the probability function of non-specific binding. The later function might be more complex and represent the technical background noise and natural biological noise determinate by large number of non-specific "low avidity" TF-DNA binding events. Parameter *s *is the relative frequency of specific bindings in (1). 0<s<1. m = 0, 1, 2,... The model (1) could be approximated by the following empirical probability distribution function:(2)

where  is the empirical probability distribution function of specific binding,  is the empirical probability of non-specific binding. The parameter *α *could be estimated as the fraction of the extended DNA fragments uniquely mapped on the genome and belonged to the true loci having specific TFBS. 0 <*α *< 1. Figure 2 shows the examples of non-normalized empirical frequency distribution of TF BEs (e.g. Nanog TF-DNA data) which after normalization to 1 can modeled by (2).

Due to sequence read sampling, the frequency distribution (1) is considered as *scale-dependent *and *skewed *functions [20,32], i.e. when the sample size, *M*, increasing, the shapes of the noise and specific frequency distribution functions are changed correspondently with library size. We model the non-specific avidity distribution function *P*_*ns *_in (1) by an exponential function distribution with continuous exponent parameter as a decay function of sample size *M *[9,11].

### The effect of a limited sample size, complex background noise, critical cut-off values, and the specificity and sensitivity of ChIP-seq assays

If one has prior knowledge of the sets of all TFBSs and of all sequences not bound by a given TF in the genome, then conventional calculation of the specificity and sensitivity of genome-wide TF BEs is straightforward. However, in the absence of such knowledge, one needs to rely on statistical analysis of data-driven physical models and computational estimates using available highly-noisy and incomplete DNA fragment samples [9,12].

A significant amount of non-specific genomic DNA fragments (background noise) is always present in the inmmunoprecipitated DNA material of any ChIP-derived dataset [9,12]. Some non-specific DNA might be easily filtered out after computer mapping of the DNA fragments on the genome. In larger library, the number TF specific loci could be increased. However, background (or noise) genomic DNA fragments could non-uniformly located in the genome and thus false clustered should be occurred in any region of the empirical frequency distribution [7,9,12,13,15].

The following basic statistical tasks are becoming imperative: (i) specificity of the library, i.e. to identify statistically significant TFBSs and count their number at the given confidence level, *t*, of BEs; (ii) power of the library, i.e., to identify "true" specific BEs which are present in the noise-enriched subset of relatively low read counts (0<*m*<t) in the library and (iii) sensitivity of the detection, i.e. the number of "lost" BSs which are available for TF binding in the given cells at the given condition, but were not detected due to a limit of the TF library size and the technical implementation.

We analyze these problems via probabilistic modelling, goodness-of-fit analysis and computational modelling of non-specific and specific BEs loci for a given TF in the ChIP-seq library. This analysis is used to quantify avidity of binding events of eleven TFs studied in the genome of mouse stem cells.

For a given TF library, let *N *denote the sum of two subsets of BEs:(3)

where *N*_1 _is the number of observed 'noise-rich' TF- DNA binding loci, having relatively low/moderate potential of TF binding avidity and *N*_2 _is the number of observed 'specific- rich' TF-DNA binding DNA loci, having a relatively high potential of TF binding avidity. The parameter *t *is the TF-DNA binding specificity threshold value.

To quantify specific and non-specific BEs, we could separate the uniquely-mapped ChIP-seq DNA fragments on two subsets by the following:(4)

where *M*_1_is the number of ChIP-seq DNA sequences which observed in the subset of 'noise-rich' and non-reliable TF-bounding DNA loci, *M*_2 _is the number of ChIP-seq DNA fragments which are observed in subset of reliable specific and TF-bounding DNA loci.

For a given TF library, let  denote the total number of specific TF-DNA binding loci in the ChIP-seq library. A set of specific TF-DNA binding loci could be split in two subsets by the following:(5)

where,  is the estimation of the number of specific BEs at value *m*.  is the estimate of the number of TF-DNA binding loci at m≥t. is the estimate of the number of specific TF-DNA binding loci at m<t.

To quantify avidity specific BEs and to estimate parameter *α *in (2), we could estimate the number of ChIP-seq DNA fragments in the high confidence loci, , and separate this number on two numbers by the following:(6)

Using (6), the weight parameter *α *in (2) can be estimated by the following:(7)

Parameter *t *is an unknown threshold value of a random variable *X *domain separating the domain on two sub-domains a binding specificity level defined by the following:(8)

where  and  were defined in (2). Let  denote an estimate of the total number of BEs in the entire genome in a given cell population at a given experimental conditions (e.g. genome of mouse embryonic stem cell line E14 at given treatment). We defined  as the following(9)

where the number of non-detected TFBSs, *N*_0 _. Then, the sensitivity of the ChIP-seq assay could be estimated by the following:(10)

where  is estimated in a domain of m-value of ChIP-seq library (*m* = 1, 2, 3,..., *J*).

### Truncated Generalized Discrete Pareto (TGDP) function and estimation of ,  and 

To quantify the empirical frequency distribution of the number of ChIP-seq TF-DNA BEs and to estimate ,  and , we model the probability function  of specific binding in (1) using the truncated GDP (TGDP) function, which could be considered as a good limiting approximation of many random evolution models [20]. The GDP probability function is described as the following(11)

where the random variable *X *is the number of BEs (*m *= 1, 2,... *J*), *f *(*m*; *k*, *β, J*) is the probability that a randomly chosen specific loci has exactly *m *BEs. The *f *involves two parameters, *k*, and *β*, where *k *> 0, and *β *> -1; the normalization factor *ζ *is the generalized (due to *β *> -1) and truncated (due to *J *< ∞) Riemann Zeta function value [33]:(12)

The continue parameter *k *characterizes the skewness of the probability function; the continue parameter *β *characterizes the deviation of the GDP distribution from a simple power law. *J *denotes the maximum observed number of BEs and used as an empirical parameter of the model (11)-(12). This parameter in scale-dependent cases is positively correlated with the sample size *M *[20]. Since in log-log plot the truncated function (11)-(12) exhibits systematic change of its shape when the sample size *M *is changed [20], the model could be co-called the empirical *scale-dependent *TGDP model [34].

When only the tail of the GDP is available for analysis, the double-truncated GDP function(13)

where(14)

could be used for quantification of empirical distributions. In this case(15)

Note, if the truncated distribution fits well to the left tail of the mixture distribution (e.g at m ≥ t), then  ≈ *N*_2_, and thus the number of specific BSs in TF ChIP-seq data can be estimated by(16)

### Fitting and back-extrapolation method for TGDP function

A noise background BEs could mask the specific moderate to low avidity TFBSs. It is important to estimate the numbers of specific moderate to low avidity TFBSs masked by noise background BEs in ChIP-seq data. However, these sub-sets of BEs might be not easily separated due to the distributions overlapping and sample size dependence. To estimate *t*-value at the given specificity level and the numbers of specific moderate to low avidity TFBSs associated with this t-value, we used the *fitting and back-extrapolation *method of recovery of the distributions of specific and non-specific BEs.

Briefly, our algorithm includes several steps: (i) an identification of the functions which after optimization of parameters could provide the best-fit functions approximating the left side and the right side of the empirical distribution, respectively, (ii) an extrapolation of these functions to the function overlapping region, (iii) identification of the specificity threshold t, (iv) an estimation of the weight parameter *α *in (2), (v) final correction of the estimated parameters using complete model, (vi) restoration of the values  and  based on the extrapolation method applied to the best-fit distribution functions. To fit the distribution functions we used optimization criteria and methods reported by [20,35]. We used also the non-linear regression tools of Sigma-Plot software (Version 11).

### Kolmogorov-Waring distribution function: an explanatory model of TF-DNA binding-dissociation process

In ChIP-seq experiments, short DNA sequence tags are randomly chosen and consequently aggregated onto genome clusters in the result of sampling of the tags derived from a large but finite number of a ChIP-seq dataset. What kinds of exploratory models could be used to quantify forming of ensemble of TF clusters bound on the genome DNA?

We [7,9,12,36] have shown that the tail of the empirical distribution function of TF-DNA BEs could be approximated by a skewed truncated Pareto-like function. This function is sample size dependent. Due to a limited number of sequence reads, some true TFBSs, *N*_0_, which are physically available for TF binding in the given cells at the given condition cannot be detected in a TF ChIP-Seq library, even if the noisy BEs are fully suppressed. In ChIP-based experiments, the number of non-detected TFBSs, *N*_0_, might be larger than the number of reliably detected specific TFBSs, *N*_2 _[11].

In general, an estimation of *N*_0 _within obtained from essentially incomplete samples is a very difficult statistical task [37]. First, this is due to (i) a large number of the real low- and moderate- avidity TFBSs (subset of real 'hidden' BEs, , indicated by the extrapolation curve on Figure 2A-B) which provide only a minor admixed part of BEs in the histogram associated with noise-enriched BEs found in ChIP-seq data. Second, a fraction of the rare BEs (subset *N*_0_) which is not presented in the observed dataset could be estimated if an appropriate physical-mathematical model is used. Below, we present a stochastic model of TF-DNA binding and dissociation together with the a method which allow us to estimate not only , but also *N*_0_.

The GDP model (8)-(9) can provide a good approximation of the empirical data; however this model does not allow estimating *N*_0 _in a given assay. Eq(8) can be a good approximation of the Waring probability function [20,35,38], which could count explicitly a probability of non-observed events. This function has been considered as an adequate distribution function derived from several explanatory stochastic process models including sampling genera from a heterogeneous biological population [38] and the aggregation process of particles [39]. The Waring probability function can be considered as a special steady-state solution of the stochastic birth-death processes called Kolmogorov-Waring process [20] arising in 'omics' data analyses. In particular, this model has been used for the modelling of protein domains in molecular evolution [20,35]and the sampling of SAGE gene expression tag profiles [28].

We assume that K-W function could be considered as an exploratory stochastic model of the evolution of specific TF-DNA binding and use the model to estimate *N*_0_. We assume that an evolution of specific TF-DNA interaction in the genome can be considered as stochastic binding and dissociation events while taking into account two binding and two dissociation transition probabilities. For binding, we consider the preferential attachment process (due to the specific binding potential between TF and DNA) and the Poisson process (non-specific potential). Similar two processes but with different intensities are assumed for detachments transitions (Figure 1D).

Let *p*_*m*_(*t*) = *P*(*D*_*t *_= *m*) denote the probability function associated with the random TF-DNA binding-dissociation process {*D*_*t*_, *t *≥ 0} (Figure 1D). Then the rate of the probability functions *p*_*m *_(*m *= 0, 1, 2...) of the number of TF-DNA BEs could be described by the forward Kolmogorov differential-difference equations:(17)

where *m *= 1, 2,.... The initial probabilities *p*_*m*_(0) ≥ 0 (*m *= 0, 1, 2,...) follow to the condition . Probably in the most evolving near steady-state, the random binding and dissociation processes of TF are kept near the equilibrium. This equilibrium solution can be written explicitly by stating *dp*_*m*_/*d*_*t *_= 0; *m *= 0,1,... in (17)-(18) as(19)

At *m *→ ∞, a necessary and sufficient condition for the existence of the non-trivial stationary solution (19)-(20) is provided by convergence of the series(21)

where . This condition exists when starting from some *i *= *i*_*c *_the condition *η*_*i *_≤ *ν *< 1 takes place for all *i *≥ *i*_*c *_(i.e. on the right tail of the frequency distribution).

Using (19)-(20) we can obtain the non-zero limiting probability function for the random process *D*_*t*→∞_:(22)

If we assume that the limiting probability distribution  exists, then all *dp*_*m*_/*d*_*t *_(*m *= 1, 2,...) would necessarily converge to 0 as *t *→ ∞ and we can obtain:

For our application purposes, we will consider the binding-dissociation process as a random process such that the intensity rates are the linear functions of the event *m*:(23)

and(24)

(*m *= 0, 1, 2,...), where the model parameters are defined by the following conditions . Hence, during an interval (*t*, *t *+ *h*) where *h *is infinitively small, we assume that there are four independent processes: the spontaneous TF binding on and dissociation from a given specific TFBS DNA locus, with constant intensities  and , respectively, and the TF binding on and dissociation from a specific TFBS DNA locus with the intensities proportional to the number of TFs already attached to the specific TFBS  and  respectively.

For steady-state distribution we could estimate three parameters of the steady-state random process. Let us denote . Let us also denote factorial power *z*^[*m*] ^= *z*(*z *+ 1)...(*z *+ *m *- 1), where m = 0, 1, 2,...; *z*^[0] ^= 1. Using (17)-(18) and (21)-(22), we can obtain the unique limiting probability function for the process (17)-(18) with the intensities given by (23) and (24):(25)

*m *= 0, 1, 2,.... Γ (*x*) is the Gamma function, and *B*(*x*) is the Beta function [38]. The (25)-(26) is K-W probability function [20]. When the preferential attachment and the preferential dissociation rates are equalled (), then we have the Waring distribution function [38].

The probability function (25)-(26) has the probability generating function [35]

where _2_*F*_1_(*a*, 1; *b *+ 1; *θ*) is the hypergeometric Gauss series [33]

at *α *= *a*, *β *= 1, *γ *= *b *+ 1.

In this work we will considered the following practically important conditions:

Then the limiting probability function is

and the probability

*where **and m *= 0, 1, 2,...; *p*_0,0 _≡ *p*_0_.

At near steady-state of such a binding-dissociation stochastic process, the K-W function can be simply calculated via the following simple recursive formula:(27)

where *m *= 0, 1, 2, ... the other three parameters *a, b *and *θ *are unknown parameters. Importantly, the K-W probability function allows us to estimate the value *p*_0 _which gives the fraction of undetected (un-observed) events in a given ChIP-seq experiment.(28)

where _2_*F*_1 _is the hypergeometric Gauss series [33].

Specifically, if *b *>*a *> 0 and *θ *→ 1 - 0, then(29)

Eq(19) can be used for extrapolation of K-W model up to *m *= 0 (unobserved number of BEs). Than by the following recursive formula (18), we can estimate the frequency of BEs at each value m (*m *= 1, 2, 3....).

If *p*_0 _> 0, we can define the zero-truncated limiting probability distribution  as(30)

where *m *= 1, 2, 3...

Using this formula, we can prove the following useful approximation:

If *a → *0+; *θ *→ 1; *b *> 0, then(31)

where *B*(*b*+1, *m*) is the Beta function.

The expressions (28) - (31) can be used for the quantitative analysis of the distribution of ChIP-seq derived TF-DNA binding events and for the calculation of *p*_0 _. Note that (27)-(28) provide more accurate estimates when a fraction of reliably detected TFBSs in ChiIP-seq assay becomes larger.

### Double-Truncated K-W Distribution and fitting of the ChIP-Seq TF-DNA binding distribution

The estimation of parameters in the general multi-parameter families becomes problematic when the number of unknown parameters increases. However, the three, two- or one- parameter family distributions (27)-(28) could be feasibly fitted to the empirical distributions.

In order to apply the probability function (27)-(28) to the data, let us assume that the domain of the random variable X is doubly truncated, e.g. the random variable X is restricted to the range *m *= *t*, *t *+ 1,..., *J*(*t *> 0, *J *< ∞). The probability function of the resulting truncated distribution can be re-normalized by the following(32)

This probability function corresponds to a common situation for ChIP-seq data analysis in which the occurrence values *m*= 0 and *m *= *J *+ 1, *J *+ 2,...,∞ are not observed. As  from the left, the (32) is transformed into practically useful expression(33)

which as *J *→ ∞ can be simplified to yield .

By analogy to (32), it is easy straight forward to derive the double-truncated K-W function for any low threshold value *t *(t = 1, 2,...) of the number of TFs bound to a given TFBS, and next using the re-normalized K-W function, to estimate parameters of the double-truncated K-W function using the optimization algorithm reported in [20].

### Discrete Pareto distribution function is an asymptotic of the Warring distribution function

*If θ *= 1, *b *>*a *> 0, then  is the zero-truncated K-W distribution (32) and as *m *→ ∞:(34)

i.e. the probability distribution (16) approximates the discrete Pareto probability distribution [20,33] in the right tail of the specific case of K-W distribution.

## List of abbreviations used

(GDP): Generalized Discrete Pareto; (K-W): Kolmogorov-Waring; (TF): Transcription Factor; (TFBS): Transcription factor binding site; (ChIP): Chromatin Immuno-Precipitation; (SACO): serial analysis of chromatin occupancy; (STAGE): sequence tag analysis of genome enrichment; (SD): standard deviations; (MC): Monte-Carlo method; (BEs): binding events; (PWM): position weighted matrix; (TSS): transcription start site.

## Competing interests

The authors declare that they have no competing interests.

## Authors' contributions

VK initiated and designed this study; he analyzed the computational results and wrote the manuscript. PJ provided databasing, pre-filtering data, motif search and data analysis. OS provided computational analysis of ChIP-seq data and wrote an initial draft of the manuscript. VK provided mathematical theory and computational algorithms, biological interpretation, feedback throughout the work, and wrote the manuscript. All authors have read and approved the final manuscript.

## Definitions

ChIP-seq library: list of ChIP-seq DNA fragments uniquely mapped onto reference genome

*m*: number of ChIP-seq DNA fragments shearing a unique locus in the genome; m could represent a relative level of binding avidity of a TFBS in a given genome locus. *m *= 0, 1, 2, 3,... *J*.

*n*(*m*, *M*): number of the loci representing by *m-value *in the ChIP-seq library.

*N*: number of distinct loci counted by the peak-finding algorithm (T2G) in a given ChIP-seq library.

*M*: total number of DNA fragments uniquely mapped to the genome and counted in *N *loci (or " total DNA sequence mass").

*t*: specificity threshold

*M*_1_: number of ChIP-seq DNA sequences which observed in the subset of low/moderate avidity loci

*M*_2_: number of ChIP-seq DNA sequences in low/moderate avidity loci

*N*_1_: number of observed 'noise-likely' TF-bound DNA loci, having relatively low/moderate avidity

*N*_2_: number of observed 'specific- rich' TF-bound DNA loci having relatively high binding avidity.

*P*: probability distribution function.

*P*_*s*_: probability distribution function of specific binding.

*P*_*ns*_: probability distribution function of occurrence of non-specific binding : empirical probability distribution function

: empirical probability distribution function of TF-DNA specific binding.

: empirical probability function of occurrence of non-specific binding

: Predicted number of specific genome loci in the ChIP-seq library.

: Predicted number of specific TF-bound DNA fragments in .

 estimate of the number of specific loci in the subset of observed 'specific- rich' TF-bound DNA loci.

 estimate of the number of specific loci in the subset of observed 'noise-rich' TF-bound DNA loci.

*s*: relative frequency of specific BEs in the mixture probability function (1).

*α*: estimated fraction of specific DNA fragments representing 

: Mean of TF specific binding.

TF-DNA binding avidity: an integrative quantitative characteristic of availability of a DNA locus (e.g. TFBS and its flanking region) for a given protein (e.g. TF) binding *Sp*: specificity

*Se*: sensitivity: (1-*p*_0_)100%.

*N*_*tot*_: total number of specific TF bound loci in the genome

: model- predicted total number of specific TFbound loci in the genome

*p*_0_: predicted fraction of specific TF bound loci out of ChIP-seq library data.

## Supplementary Material

Additional file 1 **GDP function fitting and extrapolation in noisy events for Esrrb TF library**. Empirical relative frequency distribution of peak height intensities for Esrrb is fitted by GDP. Log-log plot: frequency of peak height intensity for the Esrrb library; solid circles: observed frequencies for cut off 12; solid line: best fit GDP function with parameters k = 2.40 ± 0.0778, b = 10.42 ± 0.6828. Extrapolated graph with the same parameters to get the predicted TFBSs in noise enriched binding events of library. Click here for file

Additional file 2 **K-W model fits on the observed and best-fit GDP-derived data and calculates p0**. Vertical dotted lines are representing qPCR experimental threshold and Improved Model threshold. Table 1 is representing the parameters of the K-W model fitting for all TFs. Click here for file

Additional file 3 **Fitting statistics of the GDP model to the empirical frequency distribution of binding events**. *t[k], t[b] *are *t *test value for *k*, and *b *respectively. *p[k] *and *p[b] *are the *p *values. *F *is Fisher criterion (by SigmaPlot) Click here for file

Additional file 4 **The numbers of TFBS-specific DNA fragments according to different specificity thresholds**. Observed and best-fit GDP function predicted numbers of the DNA fragments are compared. Click here for file

Additional file 5 **Mutual agreement best-fit K-W and GDP functions**. the both functions provide an accurately estimation of the number of specific ChIP-seq DNA fragments in reliably-defined TF binding sites. Click here for file

Additional file 6 **Venn diagrams of number of E-boxes co-localization in ChIP-seq defined binding loci**. A: Venn diagram of number of E-boxes positive loci found in vicinity ± 150 bp of the centre of ChIP-seq defined binding loci. B: Venn diagram of number of E-boxes positive loci found in vicinity ± 250 bp of the centre of ChIP-seq defined binding loci. Click here for file

Additional file 7 **Number of c-Myc binding loci containing E-boxes in different peak height**. A: Number of c-Myc binding loci (± 150 bp from c-Myc loci center) containing E-boxes. B: Number of c-Myc binding loci (± 250 bp from c-Myc loci center) containing E-boxes. Click here for file

Additional file 8 **Validation of ChIP-seq defined c-Myc binding loci based on localization of c-Myc binding loci, E-boxes and putative promoter of genes in mouse genome**. A: Number of c-Myc BSs containing E-boxes and not containing E-boxes around TSS ± 1 kb and Number of genes which have c-Myc BSs containing E-boxes and not containing E-boxes. B: Number of BSs which are around TSS ± 1 kb and contain E-box. The number was separated in two groups; 1: relative low avidity (peak height 7-8) and 2: moderate- and high-avidity (peak height 9+) Click here for file

Additional file 9 **Number of E-boxes found within c-Myc binding loci in different peak height**. A: Number of E-boxes found within c-Myc binding loci (± 150 bp of center). B: Number of motifs found in c-Myc binding loci (± 250 bp of center). Click here for file

Additional file 10 **TF-gene association scores**. High-, moderate-, and relatively low avidity c-Myc binding loci with multiple E-boxes in putative promoter region of Wee1, Npm3, and Fkbp5, respectively. Gene enrichment classes: I - gene enriched with binding site for Nanog, Oct4, Sox2, Smad1, and STAT3; II - gene enriched with binding site for c-Myc and n-Myc. The association score estimates distance between each pair of binding locus and gene based on genomic location of the binding locus that is closest to TSS. Click here for file
