# Supplementary figures and images for: Statistics of protein-DNA binding and the total number of binding sites for a transcription factor in the mammalian genome
Source: BMC Genomics. 2010 Feb 10;11(Suppl 1):S12. doi: 10.1186/1471-2164-11-S1-S12 (PMC2822526; doi:10.1186/1471-2164-11-S1-S12)

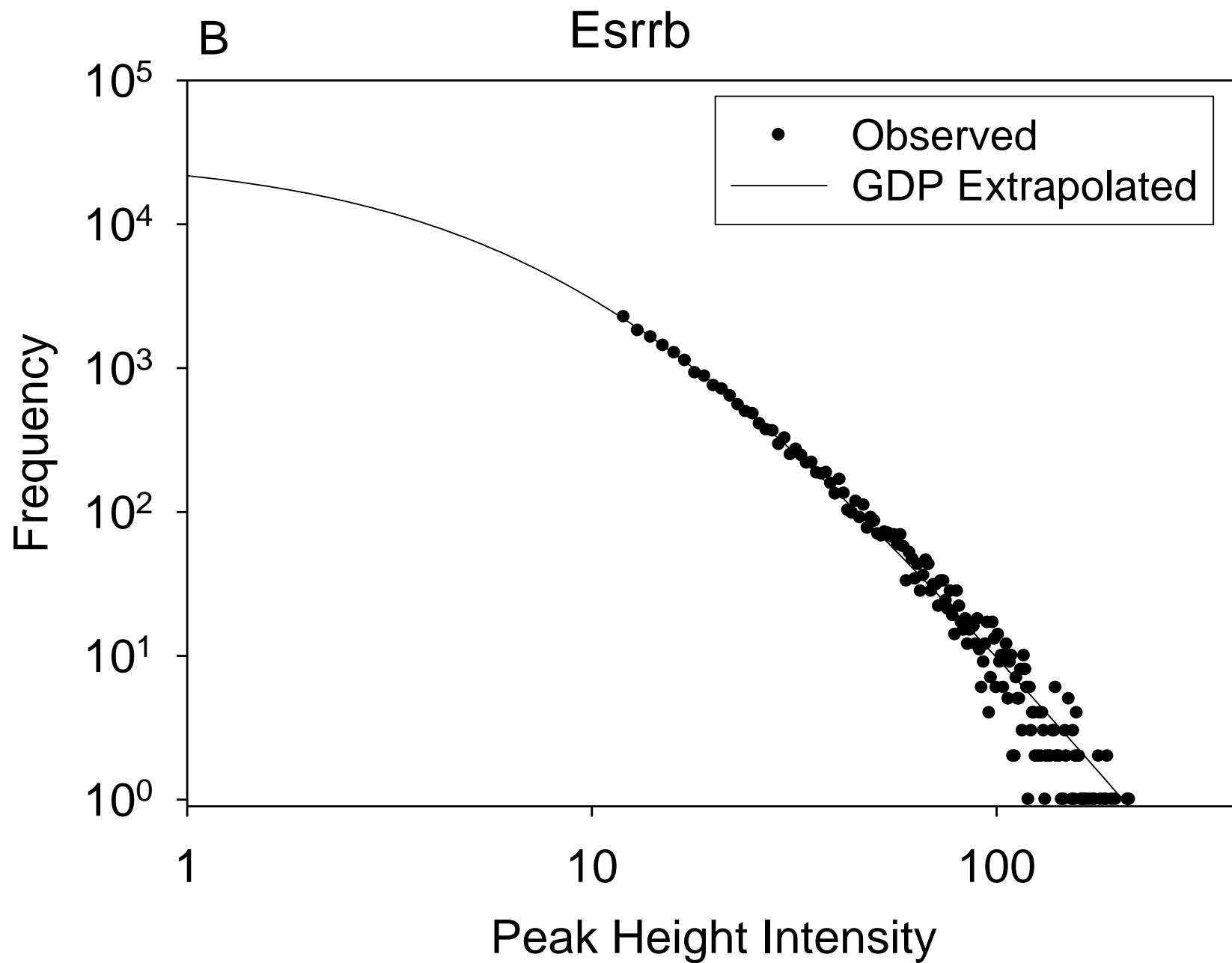

Supplement: Additional file 1 — GDP function fitting and extrapolation in noisy events for Esrrb TF library. Empirical relative frequency distribution of peak height intensities for Esrrb is fitted by GDP. Log-log plot: frequency of peak height intensity for the Esrrb library; solid circles: observed frequencies for cut off 12; solid line: best fit GDP function with parameters k = 2.40 ± 0.0778, b = 10.42 ± 0.6828. Extrapolated graph with the same parameters to get the predicted TFBSs in noise enriched binding events of library. [file 1471-2164-11-S1-S12-S1.pdf]

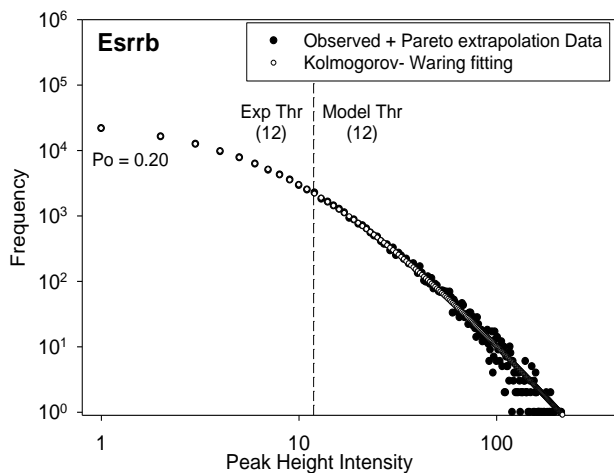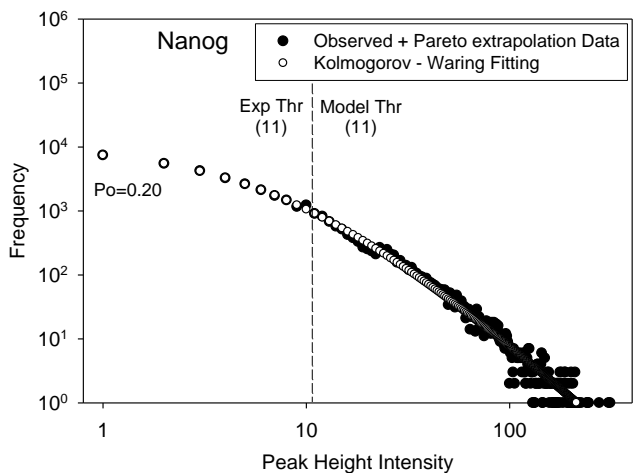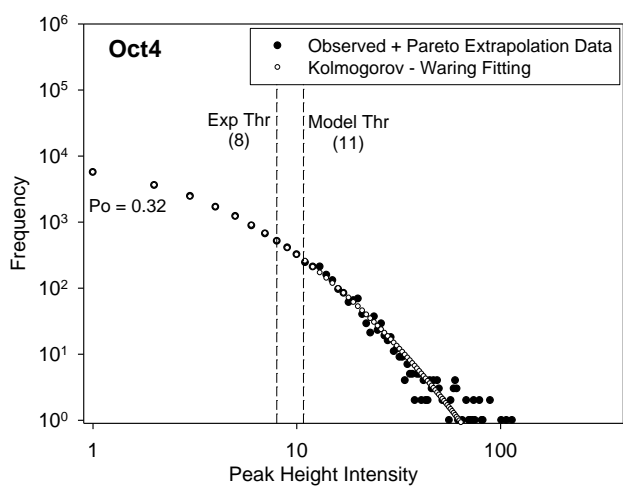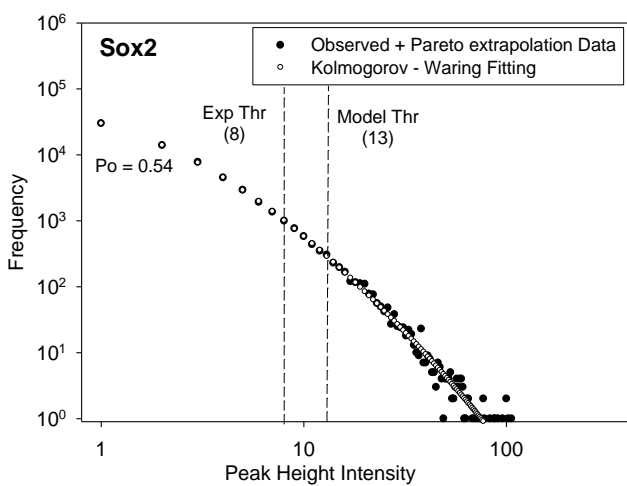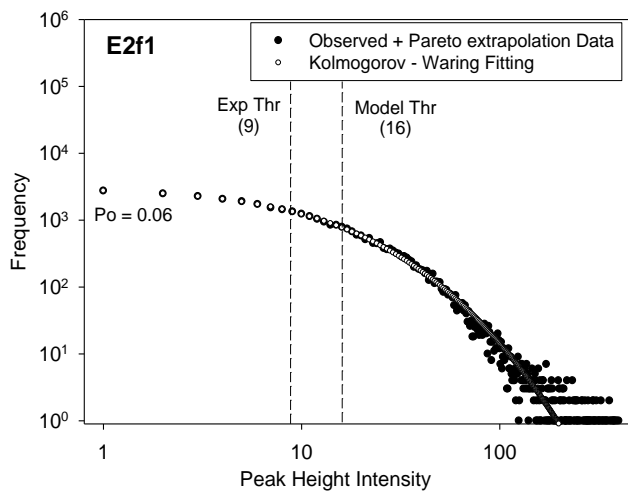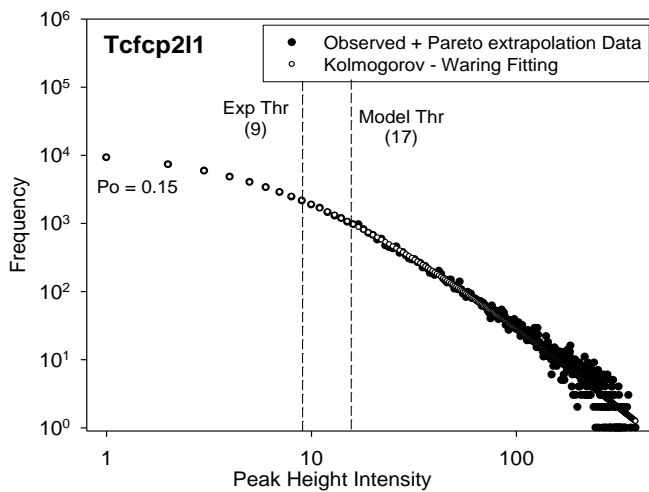

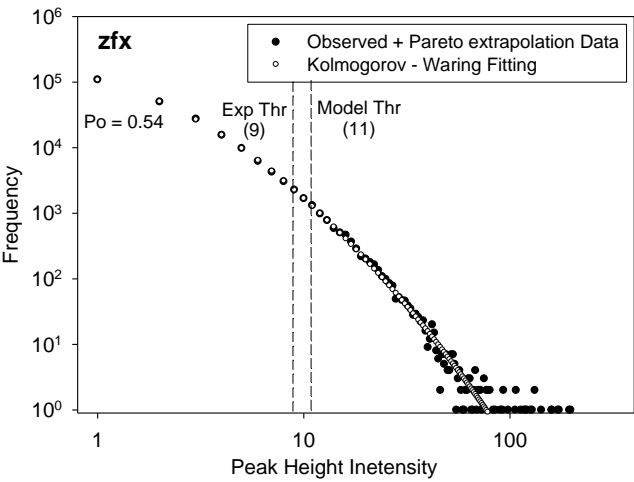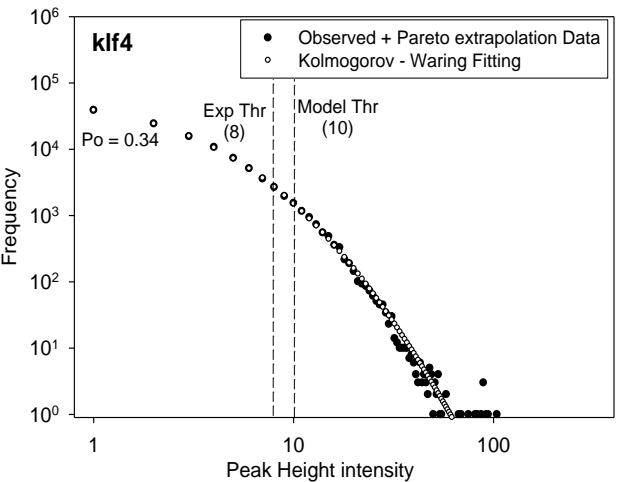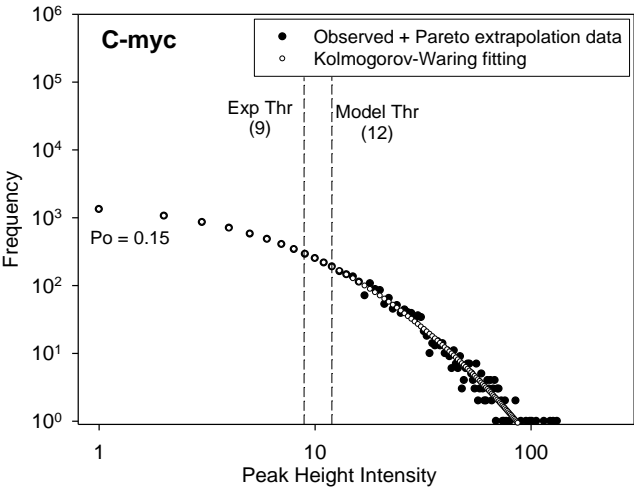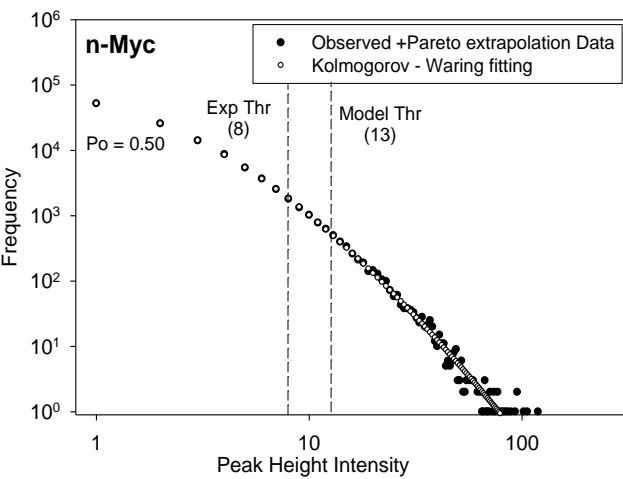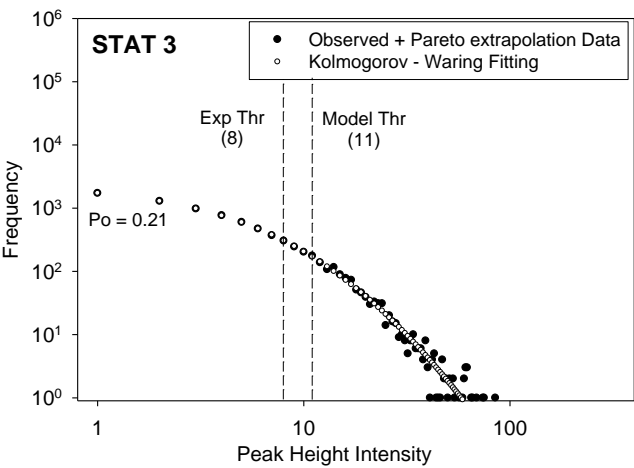

Supplement: Additional file 2 — K-W model fits on the observed and best-fit GDP-derived data and calculates p0. Vertical dotted lines are representing qPCR experimental threshold and Improved Model threshold. Table 1 is representing the parameters of the K-W model fitting for all TFs. [file 1471-2164-11-S1-S12-S2.pdf]
