# Supplementary material for: Statistics of protein-DNA binding and the total number of binding sites for a transcription factor in the mammalian genome
Source: BMC Genomics. 2010 Feb 10;11(Suppl 1):S12. doi: 10.1186/1471-2164-11-S1-S12 (PMC2822526; doi:10.1186/1471-2164-11-S1-S12)

Additional file 6

Title: Venn diagrams of number of E-boxes co-localization in ChIP-seq defined binding loci

Description: A: Venn diagram of number of E-boxes positive loci found in vicinity ±150 nt of the centre of ChIP-seq defined binding loci. B: Venn diagram of number of E-boxes positive loci found in vicinity ±250 nt of the centre of ChIP-seq defined binding loci.

A)

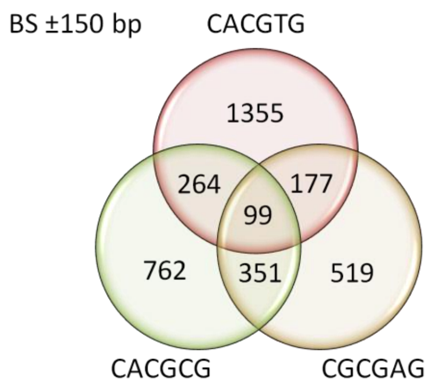

B)

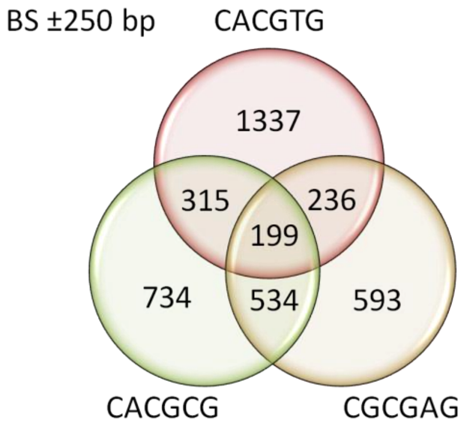

Supplement: Additional file 6 — Venn diagrams of number of E-boxes co-localization in ChIP-seq defined binding loci. A: Venn diagram of number of E-boxes positive loci found in vicinity ± 150 bp of the centre of ChIP-seq defined binding loci. B: Venn diagram of number of E-boxes positive loci found in vicinity ± 250 bp of the centre of ChIP-seq defined binding loci. [file 1471-2164-11-S1-S12-S6.pdf]
